# Nanomaterial with Core–Shell Structure Composed of {P_2_W_18_O_62_} and Cobalt Homobenzotrizoate for Supercapacitors and H_2_O_2_-Sensing Applications

**DOI:** 10.3390/nano13071176

**Published:** 2023-03-25

**Authors:** Lanyue Zhang, Shan Di, Hong Lin, Chunmei Wang, Kai Yu, Jinghua Lv, Chunxiao Wang, Baibin Zhou

**Affiliations:** 1 Key Laboratory for Photonic and Electronic Bandgap Materials, Ministry of Education, Harbin Normal University, Harbin 150025, China; 2 Key Laboratory of Synthesis of Functional Materials and Green Catalysis, Colleges of Heilongjiang Province, Harbin Normal University, Harbin 150025, China

**Keywords:** Dawson-type polyoxometalates, 1,3,5-benzylcarboxylic acid, core–shell structure, supercapacitor, H_2_O_2_ sensing

## Abstract

Designing and preparing dual-functional Dawson-type polyoxometalate-based metal–organic framework (POMOF) energy storage materials is challenging. Here, the Dawson-type POMOF nanomaterial with the molecular formula CoK_4_[P_2_W_18_O_62_]@Co_3_(btc)_2_ (abbreviated as {P_2_W_18_}@Co-BTC, H_3_btc = 1,3,5-benzylcarboxylic acid) was prepared using a solid-phase grinding method. XRD, SEM, TEM et al. analyses prove that this nanomaterial has a core–shell structure of Co-BTC wrapping around the {P_2_W_18_}. In the three-electrode system, it was found that {P_2_W_18_}@Co-BTC has the best supercapacitance performance, with a specific capacitance of 490.7 F g^−1^ (1 A g^−1^) and good stability, compared to nanomaterials synthesized with different feedstock ratios and two precursors. In the symmetrical double-electrode system, both the power density (800.00 W kg^−1^) and the energy density (11.36 Wh kg^−1^) are greater. In addition, as the electrode material for the H_2_O_2_ sensor, {P_2_W_18_}@Co-BTC also exhibits a better H_2_O_2_-sensing performance, such as a wide linear range (1.9 μM–1.67 mM), low detection limit (0.633 μM), high selectivity, stability (92.4%) and high recovery for the detection of H_2_O_2_ in human serum samples. This study provides a new strategy for the development of Dawson-type POMOF nanomaterial compounds.

## 1. Introduction

With the ever-changing energy types and demands, how to better develop and utilize clean energy and design devices for better energy storage has become the current research hotspot. Due to their lengthy cycle life and high specific capacitance, extensive attention scholars have been attracted to supercapacitors [1,2]. As a green energy storage device, they can be divided into pseudocapacitors (PCs) and electric double-layer capacitors (EDLCs) according to the charge storage mechanism [3,4]. Electrode materials are at the heart of the research on these two types of supercapacitors. The electrode materials of PCs are mostly metal oxides [5], such as RuO_2_ [6], MnO_2_ [7], WO_3_ [8] and MoO_3_ [9], which are characterized by the ability to store charges on the electrode surface through redox reactions, in addition to the formation of electric double-layers through ion adsorption. They have the advantages of a high specific capacitance and high energy density. EDLCs are mainly made of carbon materials, which have the characteristics of a good cycle stability and high electrical conductivity [10,11,12]. Supercapacitors are limited in practical applications due to defects such as a poor PC rate performance, short cycle life and low EDLC specific capacitance [13]. Therefore, developing electrode materials with excellent properties and satisfactory practical applications is a challenging task.

Polyoxometalates (POMs) are clusters of various metal oxides with promising applications in many fields, such as electrochemistry [14] and medicine [15]. Recently, POMs have been regarded as one of the most promising materials for supercapacitors and H_2_O_2_-sensing electrodes due to their reversible redox activity, high charge density and electron storage capacity. In the existing literature, hybrids of Keggin-type POMs have been studied more [16,17,18,19,20,21,22]. However, Dawson-type POMs are rarely reported. For example, Ma [23] group synthesized a novel coordination polymer (H_2_bpe)(Hbpe)_2_{[Cu(pzta)(H_2_O)][P_2_W_18_O_62_]}·5H_2_O with good electrochemical properties using a hydrothermal method in 2017. More reports are shown in Table S1. The data in Table S1 show that Dawson POMs are widely used in photocatalysis and sensing, and the highly negatively charged, oxygen-rich surface of Dawson-type POMs can enhance the charge separation and electron transmission efficiency [24,25,26], which is beneficial for improving the performance of supercapacitors and H_2_O_2_ sensors. In recent years, our group has reported several cases of Dawson-type POM compounds with an excellent supercapacitors performance synthesized using the hydrothermal method [27,28,29,30,31]. However, its large solubility in electrolytes or water and low intrinsic conductivity hinder its practical application as a solid electrode material in supercapacitors and sensing. As a new type of functional material, metal–organic frameworks (MOFs) have the advantages of chemical sustainability and a high specific surface area, and their organic ligands and metal ion properties have a great impact on the adaptability of POMs [32,33]. Therefore, immobilizing POMs on MOFs to prepare POMOFs is a good approach to improve electronic conductivity and reduce solubility to optimize their performance as solid electrode materials [34]. Dawson-type POMOFs are usually obtained using the hydrothermal method [35,36,37], but the difficulty of repetition and low yield limit their large-scale application. The grinding method is a simple and efficient method [38]. Thus, we propose a solid-phase grinding method to synthesize POMOFs, which solves the problems present in the hydrothermal method [39].

Green and pollution-free oxidant-H_2_O_2_ is widely used in production and life. High levels of H_2_O_2_ in the environment will have some harmful effects on the human body, such as causing gene damage. It is significant to realize the rapid and accurate detection of H_2_O_2_ content. Electrochemical detection methods have attracted attention due to their high sensitivity and low cost, and the main sensor materials used are nanocomposite materials and enzymes. Nevertheless, the poor stability of the two materials limits their applications [40]. Finding suitable sensor materials is one of the research hotspots of H_2_O_2_ sensors.

On this basis, the core–shell structure {P_2_W_18_}@Co-BTC was synthesized using [P_2_W_18_O_62_]^6−^, Co^2+^ and H_3_btc ligands. The synthesis was considered as follows: (I) the surface of [P_2_W_18_O_62_]^6−^ has many negatively charged oxygen atoms and it is easy for it to cooperate with transition metals to form new structures; (II) the various coordination modes of transition metal Co^2+^ give it a better performance in supercapacitors; (III) It is easy to coordinate H_3_btc with transition metals, with various coordination modes and binding sites. Based on the above considerations, a core–shell structure with Co-BTC as the shell wrapped on the {P_2_W_18_} core was synthesized using the solid-phase grinding method. Notably, the synergy between the core ({P_2_W_18_}) and the shell (Co-BTC) enhances the electronic conductivity and stability of the nanomaterial, and also provides a larger specific surface area and an abundance of active sites.

In this paper, we performed a series of characterizations on the nanomaterials, such as IR, XRD, SEM, TEM, XPS, etc. In the three-electrode system of supercapacitors, the performances of the target nanomaterial and precursors were compared, and the performance gap between nickel foam (NF) and carbon cloth (CC) as collectors and the synthesis ratio of nanomaterials were discussed. In addition, the symmetrical double-electrode test and H_2_O_2_ sensing test were performed.

## 2. Materials and Methods

### 2.1. Synthesis of CoK_4_[P_2_W_18_O_62_]

K_6_[P_2_W_18_O_62_]·15H_2_O (0.15 mmol, see Supplementary Information for its synthesis) and Co(NO_3_)_2_ (0.41 mmol) were dissolved in 10 mL of distilled water. The pH was adjusted to 4.5 with 0.1 M NaOH, and, after stirring for 6 h at room temperature, the mixture was evaporated, concentrated, crystallized and dried at 60 °C for 24 h, obtaining CoK_4_[P_2_W_18_O_62_].

### 2.2. Synthesis of Co_3_(btc)_2_

H_3_btc (0.49 mmol) and Co(NO_3_)_2_ (0.41 mmol) were weighed in a mortar, 0.5 mL anhydrous ethanol was added every hour, and the sample was ground with approximately 2~3 kg force for 3 h. The sample completely changed to light pink and was dried to obtain Co_3_(btc)_2_ (abbreviated as Co-BTC).

### 2.3. Synthesis of CoK_4_[P_2_W_18_O_62_]@Co-BTC

**Scheme 1:**
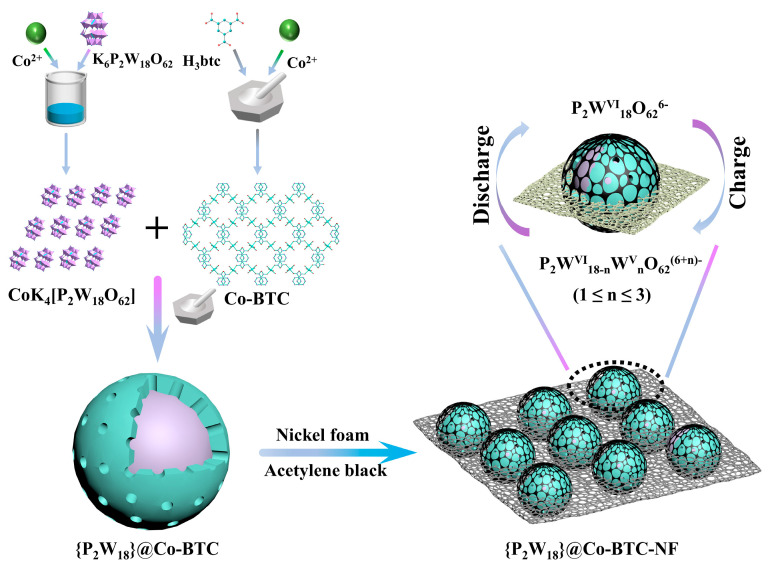
The synthesis strategies of {P_2_W_18_}@Co-BTC nanomaterial and {P_2_W_18_}@Co-BTC-NF and the working mechanism of {P_2_W_18_}@Co-BTC-NF in supercapacitors are illustrated.

The CoK_4_[P_2_W_18_O_62_] (0.10 mmol) and Co-BTC (0.30 mmol) were mixed and 1 mL of anhydrous ethanol was added every hour for 2 h. Then, the sample was ground with approximately 2~3 kg force for 2 h. The sample color gradually changed to dark pink, and was then washed three times with anhydrous ethanol and separated by centrifugation, followed by being dried in an oven at 60 °C for 24 h to obtain the nanomaterial CoK_4_[P_2_W_18_O_62_]@Co-BTC (abbreviated as {P_2_W_18_}@Co-BTC). In the same method, nanomaterials with feeding ratio of 1:1, 1:2 and 1:4 from CoK_4_[P_2_W_18_O_62_] to Co-BTC were synthesized, expressed as {P_2_W_18_}@Co-BTC-1, {P_2_W_18_}@Co-BTC-2 and {P_2_W_18_}@Co-BTC-3, respectively. For the synthesis strategy, see Scheme 1.

## 3. Results and Discussion

### 3.1. Characterization

**Figure 1:**
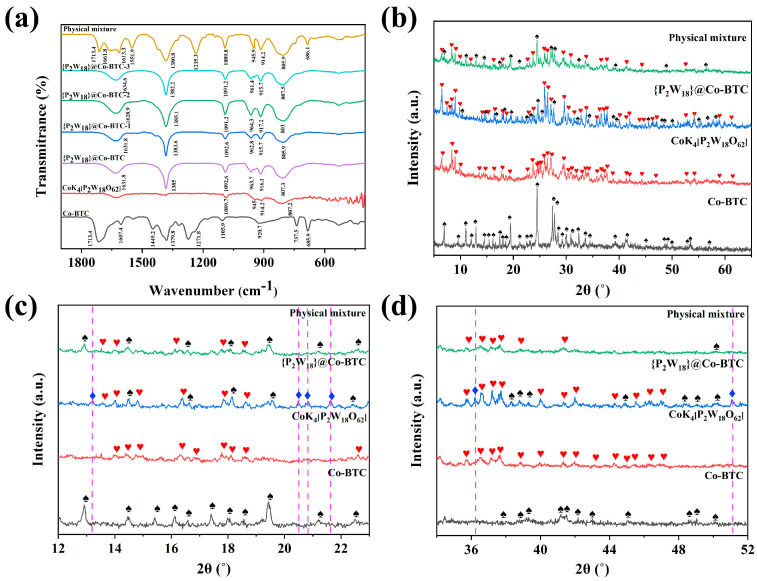
(**a**) The IR of Co-BTC, CoK_4_[P_2_W_18_O_62_], {P_2_W_18_}@Co-BTC-n (n = 0, 1~3) and physical mixture; (**b**) the XRD of Co-BTC, CoK_4_[P_2_W_18_O_62_], {P_2_W_18_}@Co-BTC and physical mixture; (**c**,**d**) the identification of the new diffraction rays (♣, ♥ and ♦ respectively represent the characteristic peaks of Co-BTC, CoK_4_[P_2_W_18_O_62_], {P_2_W_18_}@Co-BTC).

In order to determine the structure of the nanomaterial, a series of characterizations (IR, XRD, TG, SEM, etc.) were performed on the {P_2_W_18_}@Co-BTC. Figure 1a (part) and Figure S1a (ensemble) show the IR diagram of Co-BTC, CoK_4_[P_2_W_18_O_62_], {P_2_W_18_}@Co-BTC-n (n = 0, 1~3) and the physical mixture. The absorption peak near 3400 cm^−1^ belongs to the O-H peak of H_2_O. The absorption peaks at 1089.7 cm^−1^, 945.0 cm^−1^, 914.2 cm^−1^ and 807.2 cm^−1^ in CoK_4_[P_2_W_18_O_62_] correspond to the *ν*_as_(P-O_a_), *ν*_as_(W-O_t_), *ν*_as_(W-O_b_-W) and *ν*_as_(W-O_c_-W). Compared with K_6_[P_2_W_18_O_62_] [41], the introduction of Co^2+^ induces the shift in characteristic peaks, explaining the synthesis of CoK_4_[P_2_W_18_O_62_]. For Co-BTC, the absorption peak at 685.9 cm^−1^ belongs to the stretching vibration of the Co-O [42]. The absorption peaks exhibited at 1379.8 cm^−1^ and 1607.4 cm^−1^ belong to the vibrational characteristic peaks of asymmetric *v*_as_(C-O) and symmetric *v*_as_(C-O) in the carboxyl groups, these characteristics peaks being consistent with those of H_3_btc [43]. In addition, the absorption peak at 1714.0 cm^−1^ generated by the vibration of the non-ionized carboxyl group indicates that Co^2+^ binds to BTC instead of H, showing that Co-BTC can be successfully synthesized by the solid-phase grinding method. The four characteristic peaks at 1092.6 cm^−1^, 962.8 cm^−1^, 915.7 cm^−1^ and 805.9 cm^−1^ in {P_2_W_18_}@Co-BTC belong to the ν_as_(P-O_a_), ν_as_(W-O_t_), ν_as_(W-O_b_-W) and ν_as_(W-O_c_-W) in CoK_4_[P_2_W_18_O_62_], respectively. The ν_as_(W-O_t_) moved by 17.8 cm^−1^, indicating that the terminal oxygen of {P_2_W_18_} has a strong coordination effect with Co-BTC. Furthermore, the two peaks at 1631.8 cm^−1^ and 1385.0 cm^−1^ are asymmetric and symmetric *v*_as_(C-O) peaks in Co-BTC, confirming the synthesis of {P_2_W_18_}@Co-BTC. The physical mixture contains characteristic peaks of CoK_4_[P_2_W_18_O_62_] and Co-BTC. Compared with the IR spectrum of {P_2_W_18_}@Co-BTC, it is clear that the peaks of Co-BTC and CoK_4_[P_2_W_18_O_62_] in the physical mixture have no offset. Moreover, the characteristic peaks of {P_2_W_18_}@Co-BTC-1-3 clearly illustrate their successful synthesis, but with different peak intensities compared to {P_2_W_18_}@Co-BTC.

Figure 1b shows the XRD patterns of Co-BTC, CoK_4_[P_2_W_18_O_62_], {P_2_W_18_}@Co-BTC and the physical mixture. The diffraction peaks of the physical mixture are in perfect agreement with the CoK_4_[P_2_W_18_O_62_] and Co-BTC. {P_2_W_18_}@Co-BTC-n contains characteristic peaks of CoK_4_[P_2_W_18_O_62_] and Co-BTC, where the peaks are slightly shifted to match the degree of CoK_4_[P_2_W_18_O_62_], which is higher. We guessed that the diffraction peaks of CoK_4_[P_2_W_18_O_62_] in {P_2_W_18_}@Co-BTC should mask some peaks of Co-BTC (Figure S1b). In addition, {P_2_W_18_}@Co-BTC produced new diffraction peaks at 13.22°, 20.48°, 20.84°, 21.64°, 36.24° and 51.06° (in Figure 1c,d), determining that CoK_4_[P_2_W_18_O_62_] cooperated with Co-BTC to form the target synthesis product [44]. New phases also exist in {P_2_W_18_}@Co-BTC-1-3 (♦ in Figure S1b), confirming that {P_2_W_18_}@Co-BTC-1-3 are all synthesized as new nanomaterials. Moreover, {P_2_W_18_}@Co-BTC produced more new phases and sharper peaks, illustrating that this ratio (1:3) has good crystallinity and is the best ratio for the synthesis of nanomaterials.

**Figure 2:**
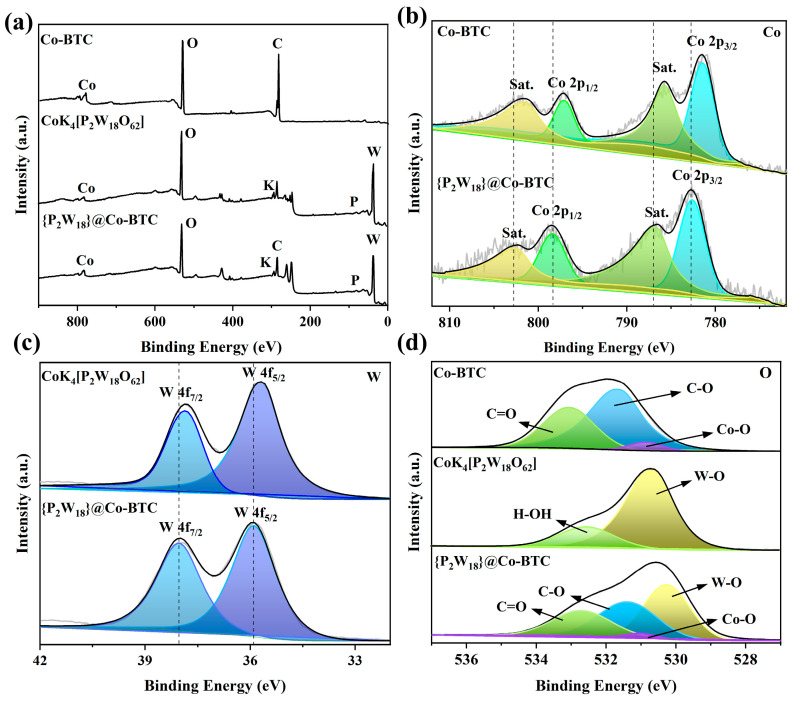
The full XPS (**a**) and O_1s_ spectra (**d**) of {P_2_W_18_}@Co-BTC, CoK_4_[P_2_W_18_O_62_] and Co-BTC; (**b**) Co_2p_ spectra of {P_2_W_18_}@Co-BTC and Co-BTC; (**c**) W_4f_ spectra of {P_2_W_18_}@Co-BTC and CoK_4_[P_2_W_18_O_62_].

XPS further verified the existence and valence of the constituent elements of the nanomaterial. The XPS test results of {P_2_W_18_}@Co-BTC, CoK_4_[P_2_W_18_O_62_] and Co-BTC are shown in Figures S2–S4 (see supplementary information for discussion). Figure 2a shows a full spectral comparison of CoK_4_[P_2_W_18_O_62_], Co-BTC and {P_2_W_18_}@Co-BTC, showing the presence of each element in the three compounds, confirming the purity of the compounds. In the Co_2p_ spectrum of {P_2_W_18_}@Co-BTC (Figure S2d), two major peaks of Co_2p1/2_ (798.4 eV) and Co_2p3/2_ (782.6 eV) along with their corresponding satellite peaks (802.4 eV and 786.6 eV) are exhibited. According to related research [45], the signal of 782.6 eV on the low-binding-energy side is assigned to Co(II), which means that the main existing state of Co in {P_2_W_18_}@Co-BTC is the Co(II) oxidation state. Compared with the four peaks of Co-BTC, the four characteristic peaks of {P_2_W_18_}@Co-BTC shifted towards the high-binding-energy side, and the maximum displacement value was approximately 5 eV (Figure 2b). Figure S2e shows the {P_2_W_18_}@Co-BTC K_2p_ spectrum, showing two main peaks around 293 eV (K_2p1/2_) and 295.9 eV (K_2p3/2_). Based on the peak values of K_2p_, the main valence state of K is +1 valence [46]. Similarly, the spectrum of W_4f_ (Figure S2c) displayed two peaks at 38 eV (W_4f5/2_) and 35.9 eV (W_4f7/2_), corresponding to the W–O bonds observed in W(Ⅵ) [41]. The P_2p_ spectrum (Figure S2b) shows the peaks at 133.7 eV (P_2p1_), corresponding to P(Ⅴ). The existence of P_2p_ and W_4f_ proves that {P_2_W_18_}@Co-BTC has retained the original framework of {P_2_W_18_}. Notably, W_4f5/2_ and W_4f7/2_ in the {P_2_W_18_}@Co-BTC underwent a positive shift of ~0.3 eV compared to the W_4f_ spectrum in CoK_4_[P_2_W_18_O_62_] (Figure 2c). {P_2_W_18_}@Co-BTC has four O_1s_ peaks—530.28 eV (W-O), 530.98 eV (Co-O), 531.38 eV (C-O) and 532.68 eV (C=O)—and it can be seen from Figure 2d that the O_1s_ peak of {P_2_W_18_}@Co-BTC showed a slight low-energy shift compared with CoK_4_[P_2_W_18_O_62_] and Co-BTC. Combined with the peak migration results of Co, O and W in {P_2_W_18_}@Co-BTC, we speculate that the electron cloud of O in CoK_4_[P_2_W_18_O_62_] overlapped with the electron cloud of Co in Co-BTC, forming an interaction that weakens the force of O and W, resulting in the peak migration of Co and O being greater than that of W. These results prove the strong interaction between Co-BTC and {P_2_W_18_}, implying the successful synthesis of {P_2_W_18_}@Co-BTC [47,48]. Among these elements, carbon (C_1s_) can also demonstrate the successful synthesis of {P_2_W_18_}@Co-BTC, where we found two peaks of C-C/C=C and C=O at 284.6 eV and 288.9 eV (Figure S2g), again confirming the presence of Co-BTC in the title nanomaterial [49]. Combining IR, XRD and XPS can clearly confirm that {P_2_W_18_}@Co-BTC can be successfully synthesized using the solid-phase grinding method.

The TG of {P_2_W_18_}@Co-BTC gives the main three steps of weight loss (Figure S5). The first and second step of weight loss is between 30~400 °C, and the weight loss rate is approximately 17.85%, which belongs to the loss of water molecules on the surface and the decomposition of Co-BTC [50]. The third step of weight loss between 400~600 °C is the decomposition of {P_2_W_18_} in {P_2_W_18_}@Co-BTC, with a weight loss rate of approximately 4.92%. The final compound is decomposed into metal oxides (Co_3_O_4_, K_2_O), tungsten carbide (W_2_C) and C, similar to the literature [51]. Combining the ICP-MS (Table S2), TG and XPS results, the molecular formula of the nanomaterial was calculated as CoK_4_[P_2_W_18_O_62_]@Co_3_(btc)_2_.

**Figure 3:**
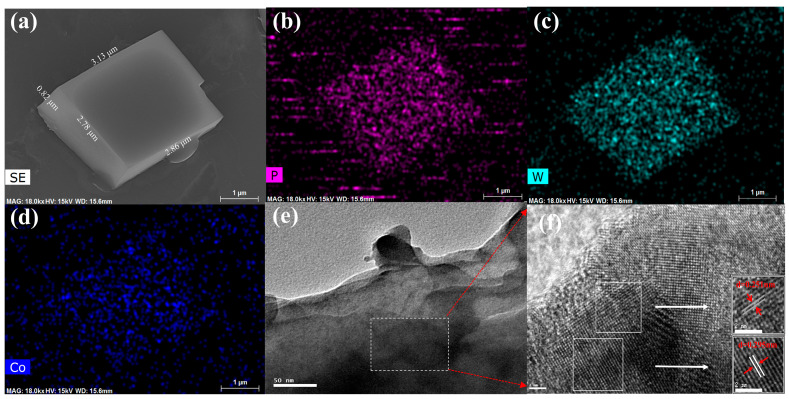
SEM images (**a**), mapping of P (**b**), W (**c**) and Co (**d**), TEM image (**e**) and the crystal plane analysis diagram (**f**) of {P_2_W_18_}@Co-BTC.

{P_2_W_18_}@Co-BTC is in the shape of a cuboid, with lengths of 3.13 μm and 2.86 μm and a width and height of approximately 2.78 μm and 0.82 μm, respectively (Figure 3a). EDS (Figure S6a) and EDX show the even distribution of C, O, K (Figure S6b–d), P, W and Co (Figure 3b–d) elements in {P_2_W_18_}@Co-BTC. Similarly, Figures S7 and S8 show the morphology of CoK_4_[P_2_W_18_O_62_] and Co-BTC as irregular massive deposits and rod-like structures, respectively, and also show the uniform distribution of each element. From the above results, in the process of combining CoK_4_[P_2_W_18_O_62_] and Co-BTC through intermolecular forces, the coordination balance of BTC and Co^2+^ changes, resulting in the deformation of Co-BTC, which makes the morphology of the nanomaterial similar to that of CoK_4_[P_2_W_18_O_62_], forming a core–shell structure with CoK_4_[P_2_W_18_O_62_] as the core and Co-BTC as the shell. The EDS results show that the molar ratio of K:P = 1.85:1 in {P_2_W_18_}@Co-BTC and K:P = 1.86:1 in CoK_4_[P_2_W_18_O_62_] was close, again verifying the purity of {P_2_W_18_}@Co-BTC. The TEM image (Figure 3e) and lattice fringe pattern (Figure 3f) of {P_2_W_18_}@Co-BTC also confirm the conjecture. Figure 3f shows that the spacings of the lattice fringe correspond to 0.295 nm (Co-BTC) and 0.251 nm ({P_2_W_18_}), and that Co-BTC can be found wrapped uniformly around the {P_2_W_18_} surface. These results demonstrate that {P_2_W_18_} is encapsulated within the metal–organic framework, providing a large specific surface area for the target product.

The N_2_ adsorption and desorption isotherms of Co-BTC, CoK_4_[P_2_W_18_O_62_] and {P_2_W_18_}@Co-BTC were tested, and the isotherms were in the shape of hysteresis loops (Figure S9a) and pore size distribution maps (Figure S9b). The specific surface area and pore size of the nanomaterials were calculated using the Barrett–Joyner–Halenda method, the specific surface areas were 12.302 m^2^ g^−1^, 11.499 m^2^ g^−1^ and 14.45 m^2^ g^−1^ and the pore sizes were 2.124 nm, 2.252 nm and 3.895 nm. As can be seen from the calculations, compared with CoK_4_[P_2_W_18_O_62_], the nanomaterials synthesized using the solid-phase grinding method not only increase the specific surface area, making the electrolyte easier to infiltrate, but also increase the pore size and improve the diffusion rate of electrolyte ions [52,53].

### 3.2. Supercapacitors Performence

Combined with the characterization results, one of the advantages of synthesizing the nanomaterial using the solid-phase grinding method is the increased specific surface area, indicating that the material has promise for use as an electrochemical electrode material and improves the electrochemical performance. The performance of the nanomaterial three-electrode supercapacitor was tested under the conditions that the electrolyte was 1 M Na_2_SO_4_ and nickel foam was the collector.

**Figure 4:**
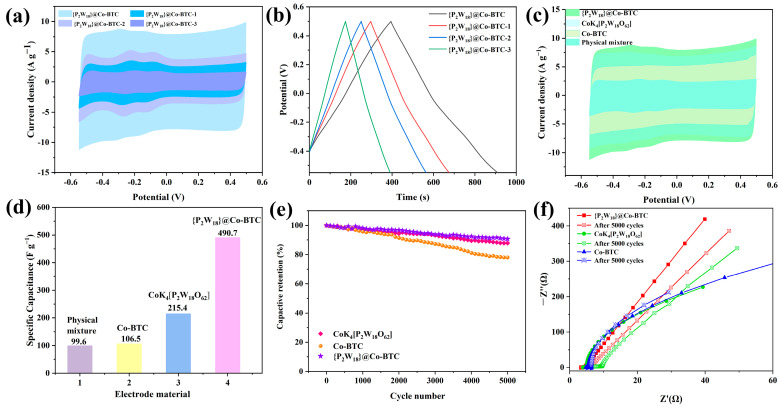
Comparison diagram of CV (**a**) and GCD (**b**) for {P_2_W_18_}@Co-BTC-n (n = 0, 1~3); (**c**) the CV comparison chart of {P_2_W_18_}@Co-BTC, CoK_4_[P_2_W_18_O_62_], Co-BTC and physical mixture in 40 mV s^−1^; (**d**) the specific capacitance of {P_2_W_18_}@Co-BTC, CoK_4_[P_2_W_18_O_62_], Co-BTC and physical mixture at 1 A g^−1^; (**e**) cycling stabilities of {P_2_W_18_}@Co-BTC, CoK_4_[P_2_W_18_O_62_] and Co-BTC; (**f**) the EIS of {P_2_W_18_}@Co-BTC, CoK_4_[P_2_W_18_O_62_] and Co-BTC before and after 5000 cycles of three-electrode system.

In order to determine the optimal ratio of nanomaterials, the effect of Co-BTC on the electrochemical performance was investigated by comparing the {P_2_W_18_}@Co-BTC-n (n = 0, 1~3) of CV and GCD. Figure 4a,b are the CV and GCD comparison diagrams of the four nanomaterials. Figure 4a shows that the ideal molar ratio of CoK_4_[P_2_W_18_O_62_] to Co-BTC is 1:3, and that the {P_2_W_18_}@Co-BTC area is the largest. According to Equation (S1), the specific capacitances of the nanomaterials with different ratios are 298.5 F g^−1^, 363 F g^−1^, 490.7 F g^−1^ and 171.6 F g^−1^, respectively, which may be related to the degree of synergy between Co-BTC and CoK_4_[P_2_W_18_O_62_] in {P_2_W_18_}@Co-BTC and the characteristics of MOF itself. The higher the content of Co-BTC, the more abundant channels and micropores of MOF, the greater the synergy with the CoK_4_[P_2_W_18_O_62_], the greater the number of active sites of {P_2_W_18_}@Co-BTC and the greater the specific capacitance. Moreover, the GCD of 1:3 (490.7 F g^−1^) is significantly greater than 1:4 (171.6 F g^−1^) due to the unbalance synergy between {P_2_W_18_} and Co-BTC. Hence, it can be inferred that the Co-BTC in {P_2_W_18_}@Co-BTC has a positive effect on its electrochemical performance, among which the nanomaterial synthesized at 1:3 performs the best.

To further investigate the supercapacitive properties of the synthesized nanomaterials under optimal conditions, the electrochemical properties of {P_2_W_18_}@Co-BTC, CoK_4_[P_2_W_18_O_62_], Co-BTC and the physical mixture were tested. Figure S10a shows the CV curve of {P_2_W_18_}@Co-BTC, showing two pairs of weak redox peaks, representing the redox process of the W atom in the [P_2_W_18_O_62_]^6−^ polyoxyanion [54]. At the same scan speed, the areas of the CV curve of {P_2_W_18_}@Co-BTC, CoK_4_[P_2_W_18_O_62_], Co-BTC and the physical mixture decrease in turn, and the pseudocapacitance of {P_2_W_18_}@Co-BTC is the largest, credited to the synergistic effect of the CoK_4_[P_2_W_18_O_62_] and Co-BTC in the nanomaterial (Figure 4c). Furthermore, the CV curves of the CoK_4_[P_2_W_18_O_62_] and Co-BTC proved their good rate performance (Figure S10b,c). Furthermore, the anodic peak current of {P_2_W_18_}@Co-BTC, CoK_4_[P_2_W_18_O_62_] and Co-BTC showed a linear relationship with the scan rate (5–40 mV s^−1^), indicating that all three species exhibit surface-controlled redox reactions, proving that they are all capacitive materials (Figure S10d) [55]. Figure S10e is the GCD comparison curve of CoK_4_[P_2_W_18_O_62_], Co-BTC, the physical mixture and {P_2_W_18_}@Co-BTC. According to Equation (S1), at a current density of 1 A g^−1^, the specific capacitances of the four compounds were 215.4 F g^−1^, 106.5 F g^−1^, 99.6 F g^−1^ and 490.7 F g^−1^ (Figure 4d). The specific capacitance of {P_2_W_18_}@Co-BTC is superior to that of a physical mixture of the same proportions because of the synergy between CoK_4_[P_2_W_18_O_62_] and Co-BTC in the POMOF nanomaterial. Meanwhile, the specific capacitance of {P_2_W_18_}@Co-BTC (490.7 F g^−1^) is higher than those reported in many literatures (see Table S3). This result may be related to the excellent electron conduction and more electron transfer pathways provided by the core–shell structure of {P_2_W_18_}@Co-BTC. From Figure 4b, the compound presence of a small iR drop can be attributed to the synergistic effect of {P_2_W_18_} and Co-BTC, and can provide a fast transport route for electrons and ions [56,57].

In order to evaluate whether {P_2_W_18_}@Co-BTC, CoK_4_[P_2_W_18_O_62_] and Co-BTC have good stability, the capacitance retention rates of the three nanomaterials at 10 A g^−1^ and 5000 charge–discharge cycles were tested, and the test results were 90.6%, 78.1% and 87.8%, respectively (Figure 4e). The CoK_4_[P_2_W_18_O_62_] anion in {P_2_W_18_}@Co-BTC has a reversible multi-electron transfer reaction, and the highly negatively charged oxygen atom on its surface increases its interaction effect with Co-BTC, thereby improving the stability of the entire structure [58]. Meanwhile, to further understand whether the nanomaterials have the potential to serve as electrodes with a good performance, EIS measurements before and after 5000 cycles were carried out, and the *R*_s_ of the three nanomaterials was calculated. *R*_s_ is the equivalent series resistance, and is a sum of the electrode, the contact resistance between the active material and the collector, and the electrolyte resistance [59]. Figure 4f and Figure S10f show the EIS spectra of the three nanomaterials before and after cycling and their magnified images in the high-frequency region, where the *R_s_* of {P_2_W_18_}@Co-BTC, CoK_4_[P_2_W_18_O_62_] and Co-BTC before cycling is 3.29 Ω, 3.96 Ω and 4.78 Ω, respectively, and the *R_s_* after cycling is 3.75 Ω, 4.26 Ω and 5.48 Ω. In the low-frequency region, the slope of the {P_2_W_18_}@Co-BTC curve before and after the cycle is the largest, meaning that the *R_ct_* of {P_2_W_18_}@Co-BTC before and after the cycle is the smallest [60]. These results demonstrate that {P_2_W_18_}@Co-BTC has ideal capacitive properties and a good rate capability, indicating that the POMOF nanomaterial with a core–shell structure reduces the dissolution of CoK_4_[P_2_W_18_O_62_] in the electrolyte and plays a positive role in the electrochemical double-layer capacitance.

**Figure 5:**
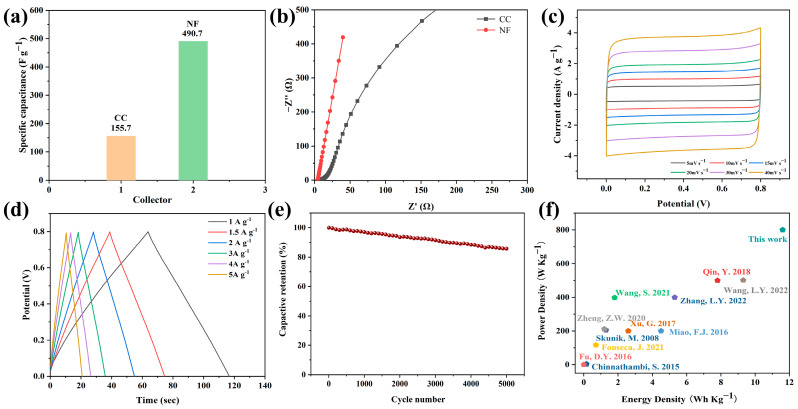
The specific capacitance (**a**) and EIS (**b**) in CC or NF as collector; the CV (**c**), GCD (**d**) and stability after 5000 cycles (**e**), Ragone plot (**f**) of {P_2_W_18_}@Co-BTC in double-electrode system [32,38,51,63,64,65,66,67,68,69,70].

In order to study the effect of different collectors on the performance of {P_2_W_18_}@Co-BTC supercapacitors, the capacitive behavior of carbon cloth collectors was tested. Figure S11a,b show that carbon cloth has stronger pseudocapacitive properties than nickel foam. As the scanning rate increases, the shape of the CV curve does not change significantly, with only a slight change in the redox peak, owing to the change in the conductivity of the electrode. Figures S10a and S11c show the CV curve of {P_2_W_18_}@Co-BTC with nickel foam and with carbon cloth as the collector. A comparison shows that, at the same scan rate of 40 mV s^−1^, the area of the CV curve of carbon cloth is smaller than that of NF, which may be attributed to the slower polarization rate of the NF electrode, which can activate more redox active sites and improve the capacitance of {P_2_W_18_}@Co-BTC. Figure 5a and Figure S11d show that the specific capacitance of {P_2_W_18_}@Co-BTC-CC (155.7 F g^−1^) is smaller than {P_2_W_18_}@Co-BTC-NF (490.7 F g^−1^), indicating that the NF electrode can significantly improve the discharge time of {P_2_W_18_}@Co-BTC, which is associated with the excellent electrical conductivity provided by the abundant pore structure of the NF electrodes. The EISs of {P_2_W_18_}@Co-BTC on CC and NF were also compared (Figure 5b), showing that the *R_s_* for CC (6.43 Ω) is larger than the *R_s_* for NF (3.29 Ω) due to the influence of the resistivity of different electrode materials. In addition, the slopes of {P_2_W_18_}@Co-BTC-CC and {P_2_W_18_}@Co-BTC-NF curves in the high-frequency region (*R_ct_*-CC < *R_ct_*-NF) may be caused by the unique porous structure of NF, allowing it to adhere more closely to {P_2_W_18_}@Co-BTC and enhance the rapid charge transfer capability at the interface of electrolyte Na_2_SO_4_ and nanomaterial NF [61,62]. Therefore, nickel foam is considered to be a more suitable electrode material for this nanomaterial.

The results show that the synthesized nanomaterial has the best performance when the collector is nickel foam and the raw material molar ratio is 1:3. To further study the value of {P_2_W_18_}@Co-BTC in practical application, an aqueous symmetric supercapacitor (ASS) (see Figure S12) was constructed with nickel foam as the collector. The CV, GCD, EIS and cycling under positive voltage (0~0.8 V) of the ASS were tested. The CV test (Figure 5c) displays that ASS is a double-layer capacitor. With an increase in the scan rate, the shape of the CV curve does not change significantly, with only a change in the current intensity, showing a higher scan rate capability. The GCD curves of ASS at current densities of 1, 1.5, 2, 3, 4 and 5 A g^−1^ were tested and are shown in Figure 5d, and, by calculation (according to Equation (S2)), the maximum specific capacitance (130.5 F g^−1^) of ASS can be obtained at 1 A g^−1^. The capacitance retention rate of ASS is 84.7% after 5000 cycles of charge and discharge at 10 A g^−1^ (Figure 5e). To further understand the electrochemical performance of ASS in practical applications, electrochemical impedance measurements were carried out (Figure S13). The internal resistances (*R_s_*) of ASS before and after cycling were 7.7 Ω and 8.7 Ω, respectively, and the resistance increased slightly after cycling, indicating that the electronic conductivity of the electrode decreased slightly. In the low-frequency region, the slope of the curve is close to 45°, indicating that ASS has relatively excellent *R_ct_* and electrochemical properties that are inseparable from the special core–shell structure of POMOF. Meanwhile, the calculated power density of ASS is 800.00 W kg^−1^ and the energy density is 11.36 Wh kg^−1^ (according to Equations (S3)–(S4)), which is higher than many previous reports (Figure 5f) [32,38,51,63,64,65,66,67,68,69,70]. The results demonstrate that {P_2_W_18_}@Co-BTC is a promising electrode material.

### 3.3. H_2_O_2_ Sensor Performance

**Figure 6:**
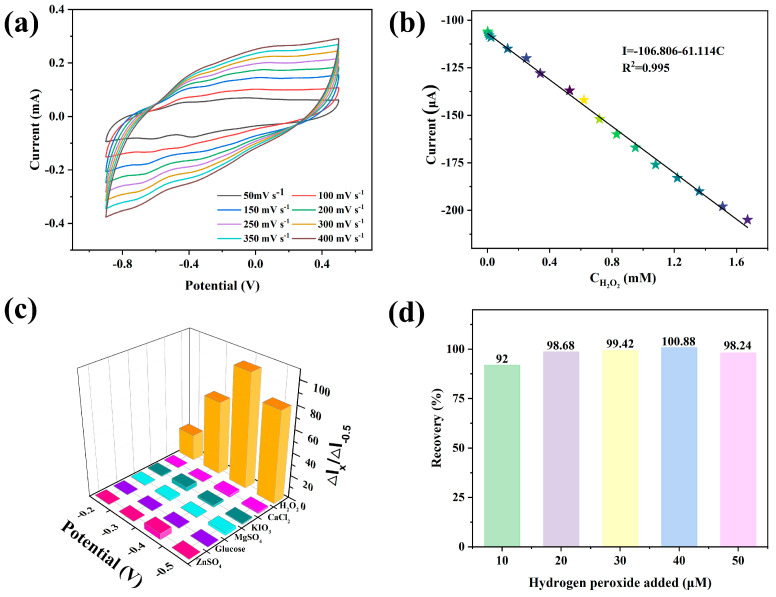
(**a**) CV curve of {P_2_W_18_}@Co-BTC-GCE at different scan rates; (**b**) calibration curve between the current response of {P_2_W_18_}@Co-BTC-GCE and the concentration of H_2_O_2_; (**c**) the effects of different interferences on the sensor response; (**d**) the recovery values of H_2_O_2_ in human serum was detected by {P_2_W_18_}@Co-BTC-GCE sensor.

Here, {P_2_W_18_}@Co-BTC synthesized by the optimal ratio was selected as the electrode material, and the glassy carbon electrode was used as the collector (referred to as {P_2_W_18_}@Co-BTC-GCE). Figure S14a is the CV curve of {P_2_W_18_}@Co-BTC-GCE at a scan rate of 50 mV s^−1^. Two pairs of redox peaks with an average peak potential of −0.216 and −0.519 V (Equation (S5)) can be observed, which are related to the occurrence of one-electron and two-electron reduction processes for the {P_2_W_18_} anion [71]. Figure 6a shows the CV curves of {P_2_W_18_}@Co-BTC-GCE at different scan rates at −0.9–0.5 V. With an increase in the scan rate, the cathodic peak shifts to negative potential and the anodic peak shifts to positive potential [72]. Furthermore, Figure S14b shows that both the oxidation and reduction peak currents of {P_2_W_18_}@Co-BTC-GCE are positively correlated with the scan rate, implying that this nanomaterial is also a surface-controlled redox process in H_2_O_2_ sensing. In order to test the catalytic activity of {P_2_W_18_}@Co-BTC-GCE, H_2_O_2_ solutions with concentrations of 0, 5, 10, 20, 30 and 40 mM were added under continuous stirring conditions, and the response current increased significantly with an increase in H_2_O_2_ concentration. In addition, the cathodic peak current also increased correspondingly, suggesting that it has a good effect on the reduction of H_2_O_2_ and good catalytic activity (Figure S14c) [73].

The sensing performance of a nanomaterial is closely related to its sensitivity and detection limit, so we performed the current–time (*i-t*) test at the maximum response voltage. Figure S14d is the *i-t* curve of {P_2_W_18_}@Co-BTC-GCE during the continuous addition of H_2_O_2_. It can be found that the increasing speed of the response current increases with the increase in H_2_O_2_ concentration [74,75]. Figure 6b is the standard curve of the current response of the title nanomaterial as a function of H_2_O_2_ concentration. According to the figure, when {P_2_W_18_}@Co-BTC-GCE was used as the electrochemical sensor to detect H_2_O_2_, the linear equations were I(μA) = −106.806–61.114 C(mM) and R^2^ = 0.995 and the detection limit was 0.633 μM (S/N = 3). The sensitivity was 8.65 mA mM^−1^ cm^−2^ and the total linear range was 1.9 μM~1.67 mM, superior to literature reports [76,77,78,79]. The linear range may be due to the large specific surface area and porosity of the POMOF core–shell structure, which facilitates the homogeneous dispersion of precursors and the transfer of electrons to the internal structure, thereby improving the reaction kinetics on the surface of the electrode material [80].

CaCl_2_, MgSO_4_, ZnSO_4_, KIO_3_ and glucose solutions were used to examine the anti-interference and selectivity of {P_2_W_18_}@Co-BTC-GCE. Figure 6c shows a comparison of the current responses of the nanomaterials and the above five interfering agents at different voltages. Two phenomena can be observed: first, the catalytic current of the nanomaterials for H_2_O_2_ is the most obvious regardless of the voltage tested; second, among the interfering substances, {P_2_W_18_}@Co-BTC-GCE has a weak current response to ZnSO_4_, and other substances respond weakly. These results indicate that {P_2_W_18_}@Co-BTC-GCE has a good anti-interference ability and selectivity [81]. Meanwhile, the CV cycling curve was tested at 40 mV s^−1^ to examine the stability of {P_2_W_18_}@Co-BTC-GCE (Figure S15). After 1000 cycles, the shape and peak value of the curve minimally change, and the retention rate of {P_2_W_18_}@Co-BTC-GCE is 92.4%, proving its excellent cycling stability.

To verify whether the sensor is promising for practical applications, real serum samples were selected for hydrogen peroxide content analysis. After diluting the serum samples 20 times with 0.1 M PBS (pH = 7.0) buffer solution, the samples of different concentrations were analyzed by the instrument, and the recovery values were calculated to be between 92.00 and 100.88% (Figure 6d), showing that {P_2_W_18_}@Co-BTC-GCE has a certain practical application prospect [82,83].

## 4. Conclusions

A {P_2_W_18_}@Co-BTC nanomaterial with a molar ratio of 1:3 between CoK_4_[P_2_W_18_O_62_] and Co-BTC was prepared using the solid-phase grinding method, which has a lower resistance, better stability and higher specific capacitance. For the three-electrode system, the specific capacitance of {P_2_W_18_}@Co-BTC with nickel foam as the collector was higher than that of CoK_4_[P_2_W_18_O_62_] (215.4 F g^−1^), Co-BTC (106.5 F g^−1^), the physical mixture (99.6 F g^−1^) and {P_2_W_18_}@Co-BTC-n, which was greater than that when carbon cloth was the collector (155.7 F g^−1^). Furthermore, compared to CoK_4_[P_2_W_18_O_62_] (78.1%) and Co-BTC (87.8%), {P_2_W_18_}@Co-BTC has the best stability (90.6%). The {P_2_W_18_}@Co-BTC was assembled into a water-symmetric supercapacitor with a success rate and energy density of 800.00 W kg^−1^ and 11.36 Wh kg^−1^, respectively. The results of H_2_O_2_ sensing also show that {P_2_W_18_}@Co-BTC-GCE has a wide linear range (1.9 μM~1.67 mM), low detection limit (0.633 μM) and high stability (92.4%), as well as a good anti-interference ability and selectivity to CaCl_2_, MgSO_4_, ZnSO_4_, KIO_3_ and glucose solutions. Moreover, sensors prepared from this material can be used to analyze H_2_O_2_ in human serum samples. In summary, the Dawson-type nanomaterials synthesized using solid-phase grinding have bifunctional properties, which provide a new strategy for the synthesis of Dawson-type POMOFs and expand their application prospects in supercapacitors and H_2_O_2_ sensing.

## Data Availability

The data presented in this study are available on request from the corresponding author.

## Supplementary Materials

The supporting information can be downloaded at https://www.mdpi.com/article/10.3390/nano13071176/s1, Figure S1: (**a**) The IR spectrum of Co-BTC, CoK_4_[P_2_W_18_O_62_], {P_2_W_18_}@Co-BTC-n (n=0, 1~3) and physical mixture; (**b**) The XRD of {P_2_W_18_}@Co-BTC-n (n=0, 1~3), Figure S2: The XPS full spectrum (**a**) and high-resolution of P_2p_ (**b**), W_4f_ (**c**), Co_2p_ (**d**), K_2p_ (**e**), O_1s_ (**f**) and C_1s_ (**g**) in {P_2_W_18_}@Co-BTC, Figure S3: The XPS full spectrum (**a**) and high-resolution of P_2p_ (**b**), W_4f_ (**c**), Co_2p_ (**d**), K_2p_ (**e**) and O_1s_ (**f**) in CoK_4_[P_2_W_18_O_62_], Figure S4: The XPS full spectrum (**a**) and high-resolution of Co_2p_ (**b**), C_1s_ (**c**) and O_1s_ (**d**) in Co-BTC, Figure S5: The TG of {P_2_W_18_}@Co-BTC, Figure S6: EDS (**a**) and EDX spectra of C (**b**), O (**c**), K (**d**) of {P_2_W_18_}@Co-BTC, Figure S7: The SEM (**a, b**), EDX (**c**) and mapping (**d**) of CoK_4_[P_2_W_18_O_62_], Figure S8: The SEM (**a**), EDS (**b**), EDX (**c**) and mapping (**d**) of Co-BTC, Figure S9: The BET (**a**) and pore size distribution map (**b**) of {P_2_W_18_}@Co-BTC, CoK_4_[P_2_W_18_O_62_], Co-BTC, Figure S10: (**a**) The CV of {P_2_W_18_}@Co-BTC in 40 mV s^−1^; (**b**) CV of CoK_4_[P_2_W_18_O_62_] and (**c**) Co-BTC at three-electrode system; (**d**) The {P_2_W_18_}@Co-BTC, CoK_4_[P_2_W_18_O_62_], Co-BTC plots of the anodic peak currents vs. scan rates; (**e**) Comparison diagram of GCD for {P_2_W_18_}@Co-BTC, CoK_4_[P_2_W_18_O_62_], Co-BTC and physical mixture; (f) The EIS of {P_2_W_18_}@Co-BTC, CoK_4_[P_2_W_18_O_62_], Co-BTC before and after 5000 cycles of the three electrodes system in the high-frequency region, Figure S11: The CV of {P_2_W_18_}@Co-BTC with CC (**a**) and NF (**b**) as collectors; (**c**) The CV of {P_2_W_18_}@Co-BTC in 40 mV s^−1^ with CC as collecter; (**d**) Comparison of GCD under the condition of current density of 1A g^−1^, CC and NF as collectors, Figure S12: The aqueous symmetric supercapacitor, Figure S13: The EIS of {P_2_W_18_}@Co-BTC before and after 5000 cycles of the symmetric double-electrode system (the insert is an enlarged view of the curves in the high-frequency region), Figure S14: (**a**) The 50 mV s^−1^ CV of {P_2_W_18_}@Co-BTC-GCE ; (**b**) Linear curve of {P_2_W_18_}@Co-BTC-GCE oxidation-reduction peak (II) current as a function of scan rate; (**c**) The CV curves of electrocatalytic reduction of H_2_O_2_ by {P_2_W_18_}@Co-BTC-GCE; (d) The *i-t* curve of {P_2_W_18_}@Co-BTC-GCE with successive additions of H_2_O_2_, Figure S15: Cyclic stability (1000 cycles) for {P_2_W_18_}@Co-BTC-GCE, Table S1: {P_2_W_18_} part of the literature summary [23,25,26,27,28,29,30,31,32,34,35,36,39,41,58,63,78,84,85,86,87,88,89,90,91,92,93], Table S2: Atomic percent (%) of corresponding samples by ICP-MS analysis, Table S3: Performance comparison of Dawson-based materials with published polyoxometalate supercapacitors [19,23,27,28,29,30,31,39,63,94,95,96,97,98,99,100,101,102,103,104,105,106].
