# Supplementary material for: Nanomaterial with Core–Shell Structure Composed of {P2W18O62} and Cobalt Homobenzotrizoate for Supercapacitors and H2O2-Sensing Applications
Source: Nanomaterials (Basel). 2023 Mar 25;13(7):1176. doi: 10.3390/nano13071176 (PMC10097129; doi:10.3390/nano13071176)
Supplement: Supplementary file 1 [file nanomaterials-13-01176-s001.zip › nanomaterials-2173439-supplementary.pdf]

nanomaterials

MDPI

Supplementary Materials

Nanomaterial with Core–Shell Structure Composed of $\{P_2W_{18}O_{62}\}$ and Cobalt Homobenzotrizoate for Supercapacitors and H_2O_2-Sensing Applications

Lanyue Zhang [1], Shan Di [1], Hong Lin [1,*], Chunmei Wang [1], Kai Yu [1,2], Jinghua Lv [1], Chunxiao Wang [1] and Baibin Zhou [1,2,*]

[1] Key Laboratory for Photonic and Electronic Bandgap Materials, Ministry of Education, Harbin Normal University, Harbin 150025, China
[2] Key Laboratory of Synthesis of Functional Materials and Green Catalysis, Colleges of Heilongjiang Province, Harbin Normal University, Harbin 150025, China
* Correspondence: lhzcc_love@126.com (H.L.); zhou_bai_bin@163.com (B.Z.)

1. Experimental section

1.1. Synthesis of $K_6[P_2W_{18}O_{62}]\cdot 15H_2O$

Dissolve 50 g of Na_2WO_4 in 100 mL of distilled water, add 42 mL of concentrated H_3PO_4, reflux for 4 h, add a few drops of H_2O_2 to remove light green, and cool the solution to room temperature. 20 g NH_4Cl was added, stirred for 10 min, suction filtered, then washed with 20 mL of 5 mol L^{-1} NH_4Cl solution. The material obtained after suction filtration was dissolved in 50 mL of distilled water at 45 °C, cooled to room temperature, and 8 g of KCl was added. The precipitate was dissolved in 50 mL of 80 °C distilled water, cooled to 15 °C and filtered, 5 g of KCl was added to the filtrate, and the crude product was obtained after suction filtration, which was dried at room temperature to obtain a yellow-green powder.

1.2. Physical mixing of the samples

Take $CoK_4[P_2W_{18}O_{62}]$ (0.4593 g, 0.1 mmol) and Co-BTC (0.1772 g, 0.3 mmol) in a small beaker and mixed with a glass bar, yielding the physical mixture.

1.3. Material and characterization methods

All reagents are commercially available and used as received without further purification. The IR of the synthesized crystals were performed on the Bruker VER TEX 80 spectrometer (Bruker, Berlin, Germany). The X-ray diffraction (XRD) patterns were obtained with a D8 Advance diffractometer with Cu-Kα radiation, and the scanning ranging from 5 to 65° (Bruker, Berlin, Germany). The morphology of the samples is analyzed by scanning electron microscopy (SEM) S-4800 (Hitachi, Tokyo, Japan). Thermogravimetric (TG) analyzer Diamond 6300 is used to measure the change of sample weight with temperature or time (Perkin Elmer, Waltham, MA, USA). The transmission electron microscope (TEM) was tested using the Tecnai G2 F20 to observe the fine structure of the sample (Thermo Fisher Scientific, San Francisco, CA, USA). X-ray photoelectron spectroscopy (XPS) is used to analyze the composition and chemical valence state of the sample surface. The testing instrument is the K-Alpha+ instrument (Thermo Fisher Scientific, San Francisco, CA, USA). The specific surface area (BET) instrument is the Autosorb-iQ type specific surface area analyzer, which analyzes the specific surface area and pore size distribution of the sample (Quantachrome, Boynton Beach, FL, USA). Inductively coupled plasma mass spectrometry (ICP-MS) was performed on an Aglient 7800 instrument for elemental analysis of the samples (Aglient, Santa Clara, CA, USA).

1.4. Supercapacitor testing

The test was carried out on CHI 660E electrochemical workstation (Chenhua Instruments Co., Shanghai, China), with 1 M Na_2SO_4 solution as electrolyte, three-electrode system and symmetrical double-electrode system were tested respectively. The test methods include cyclic voltammetry (CV), Constant current charge-discharge method (GCD) and alternating current impedance spectroscopy (EIS). The reference electrode, working electrode and counter electrode of the three-electrode system were Ag/AgCl (3 mol L^{-1} KCl), nickel foam (NF) and platinum sheet, respectively. The positive and negative electrodes of the double-electrode system are all nickel foam (NF) and have roughly the same weight.

1.5. H_2O_2 sensing test

At room temperature, a three-electrode test system was constructed on a CHI 660E electrochemical workstation. Phosphate buffer solution with pH=7 was used as the electrolyte solution. The counter electrode, reference electrode and working electrode were Pt sheet, Ag/AgCl (3M KCl), glassy carbon. Cyclic voltammetry (CV) tests were performed at different scan rates, different H_2O_2 concentrations and 1000 cycles, along with I-t curves and anti-jamming tests.

1.6. Preparation of the electrode

Nickel foam (NF) electrode: The method for preparation of nickel foam-based electrode was cut nickel foam into 1×3 cm^2, ultrasonically treated in acetone and 3M HCl for 30 minutes, washed with distilled water and ethanol, and then transferred into vacuum oven. Mix the active substance, acetylene black evenly in a 1:3 mass ratio, then drop ethanol and mix until thick. Coated nickel foam with 5 mg paste at a place of 1×1 cm^2, and after drying, pressed the nickel foam into 3s slices at 3 MPa by tablet press.

Carbon cloth (CC) electrode: The carbon cloth was coated with 5 mg paste at a place of 1×1 cm^2, then dry at room temperature.

H_2O_2 sensing glassy carbon electrode (GCE): Before testing, the GCEs were ground with 1, 0.3, 0.05 mm alumina powder and thoroughly rinsed with distilled water. The working electrode slurry was prepared by mixing acetylene black and the sample at a weight ratio of 1:1, and using 1 mL of ethanol and 3 mL of distilled water as the solvent. The well-dispersed slurry (5 μL) after sonication was dropped on the GCE surface and dried at 25 °C for 2 h to form a uniform film. 5 μL of Nafion solution was dropped on the surface of GCE and dried at 25°C.

1.7. Computational formula

The specific capacitance of the three electrode can be calculated according to the GCD curve, and the formula is as follows:

$$C = I \times \Delta t / m \times \Delta V \quad \text{(S1)}$$

where C_s (F g^{-1}) is the specific capacitance, I (A) is the current during discharge, (I/m is the current density during discharge), Δt (s) means the time of discharge, m (g) is the mass of the electrode materials, and ΔV (V) is the voltage difference between the upper and lower potential limits.

The specific capacitance of symmetrical double electrode:

$$C = 2I \times \Delta t / m \times \Delta V \quad \text{(S2)}$$

Meanwhile, the energy density and power density can be calculated in the two-electrode system, and the formula is as follows:

$$E = C\Delta V^2 / 7.2 \quad \text{(S3)}$$

$$P = E \times 3600 / t \qquad \text{(S4)}$$

In equation (S3), E is the energy density in unit Wh kg^{-1}; Formula (S4) P is the power density, in unit of W kg^{-1}.

The Average peak potential formula:

$$E_{1/2}=(E_{pa}+E_{pc})/2 \qquad \text{(S5)}$$

In equation (S5), E_{pa} and E_{pc} are anodic peak current and cathodic peak current.

The formula of H_2O_2 sensing catalytic efficiencies (CAT):

$$CAT=100\%\times[Ip\{POMOF\}H_2O_2-Ip\{POMOF\}]/Ip\{POMOF\}\} \qquad \text{(S6)}$$

Among them, Ip\{POMOF\}H_2O_2 is the oxidation peak current in the presence of hydrogen peroxide, and Ip\{POMOF\} is the oxidation peak current in the absence of hydrogen peroxide.

2. Results and discussion

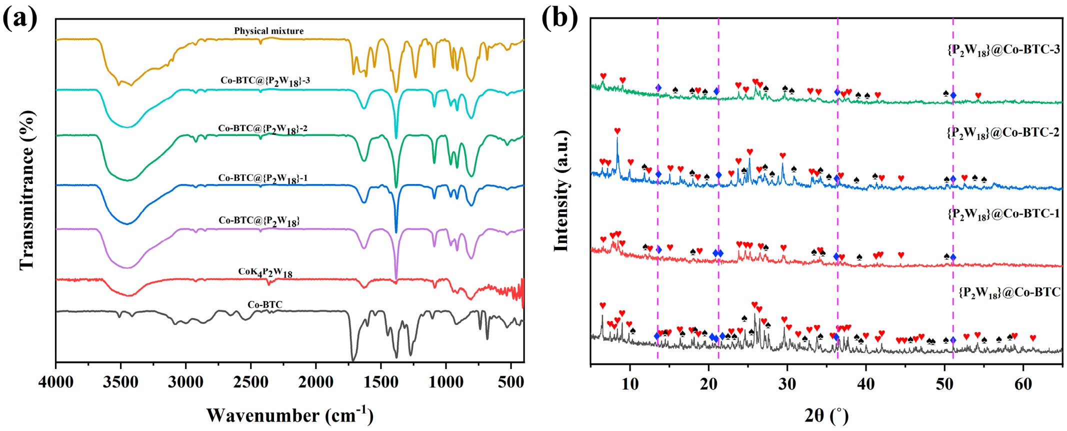

Figure S1. (**a**) The IR spectrum of Co-BTC, $CoK_4[P_2W_{18}O_{62}]$, $\{P_2W_{18}\}$@Co-BTC-n (n=0, 1~3) and physical mixture; (**b**) The XRD of $\{P_2W_{18}\}$@Co-BTC-n (n=0, 1~3) (♣, ♥ and ♦ respectively represent the characteristic peaks of Co-BTC, $CoK_4[P_2W_{18}O_{62}]$, $\{P_2W_{18}\}$@Co-BTC-n).

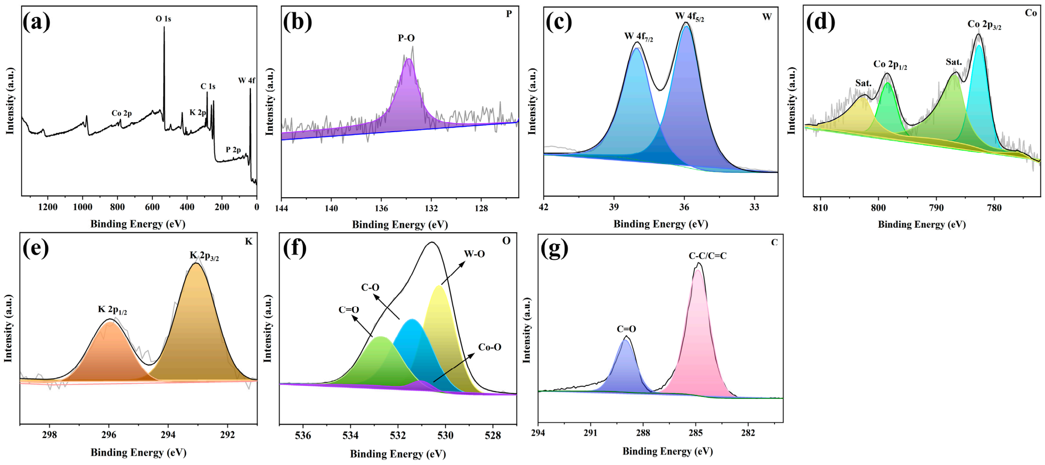

Figure S2. The XPS full spectrum (**a**) and high-resolution of P_{2p} (**b**), W_{4f} (**c**), Co_{2p} (**d**), K_{2p} (**e**), O_{1s} (**f**) and C_{1s} (**g**) in $\{P_2W_{18}\}$@Co-BTC.

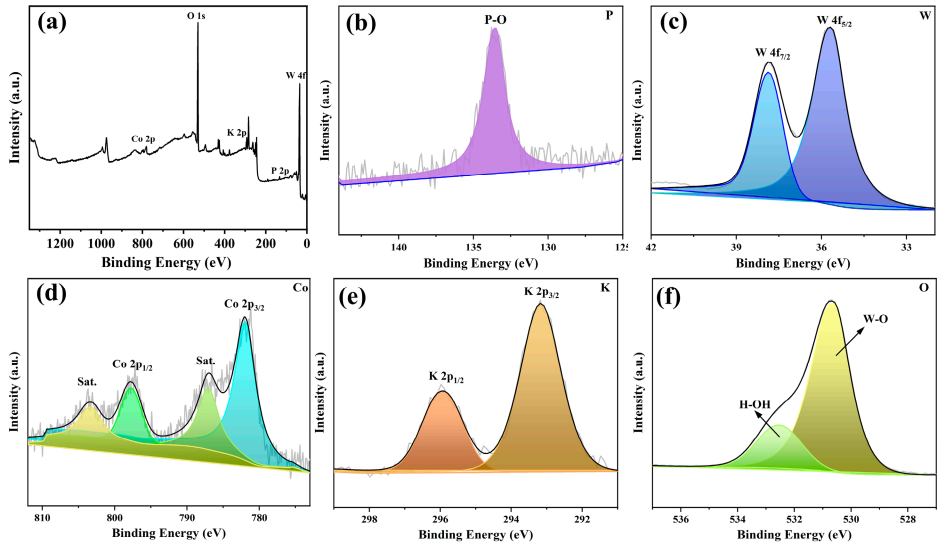

Figure S3. The XPS full spectrum (**a**) and high-resolution of P_{2p} (**b**), W_{4f} (**c**), Co_{2p} (**d**), K_{2p} (**e**) and O_{1s} (**f**) in $CoK_4[P_2W_{18}O_{62}]$. The XPS test of $CoK_4[P_2W_{18}O_{62}]$ was used to analyze its chemical valence state and composition. Figure S3a is the full spectrum. Figure S3b shows the binding energy spectrum of element P, and the characteristic peak of P-O can be observed at 133.58 eV, indicating that P exists in the +5 oxidation state. As can be seen from Figure S3C, the high-resolution XPS spectra of W_{4f} at 35.66 eV and 37.87 eV are associated with $W_{4f7/2}$ and $W_{4f5/2}$ respectively, corresponding to W^{6+}. High-resolution spectra of Co_{2p} show the presence of two major peaks, $Co_{2p1/2}$(797.72 eV) and $Co_{2p3/2}$(781.95 eV), and their corresponding satellite peaks (802.4eV and 786.6 eV), supporting the presence of Co^{2+} in the compound (Figure S3d). Figure S3d shows the K_{2p} spectrum with two main peaks near 293eV ($K_{2p1/2}$) and 295.9 eV ($K_{2p3/2}$), corresponding to K^{+1}. In addition, O_{1s} has two peaks at 530.0 eV, belonging to the binding energy of W-O and H_2O (see Figure 3e).

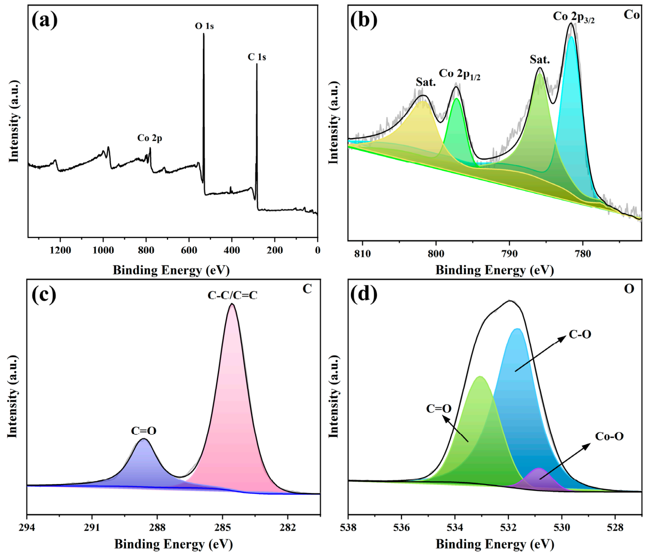

Figure S4. The XPS full spectrum (**a**) and high-resolution of Co_{2p} (**b**), C_{1s} (**c**) and O_{1s} (**d**) in Co-BTC. Three elements C, O and Co can be detected in the full XPS spectrum of Co-BTC (Figure S4a). The signal peaks at 797.1 eV and 801.4 eV belong to $Co_{2p1/2}$, and the two adjacent satellite peaks at 781.5 eV and 785.8 eV belong to $Co_{2p3/2}$(Figure S4b), demonstrating the presence of cobalt divalent ions. The C_{1s} high-resolution spectrum has two signal peaks corresponding to C-C/C=C and C=O on the benzene ring (Figure S4c). The signal peaks of O_{1s} spectrum at 530.8 eV, 531.6 eV and 533.0 eV are formed by the Co-O bond, C-O bond and C = O bond (Figure S4d).

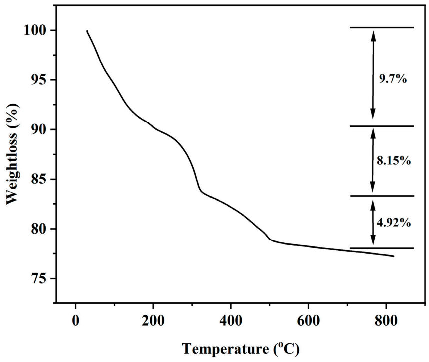

Figure S5. The TG b of {P_2W_{18}}@Co-BTC.

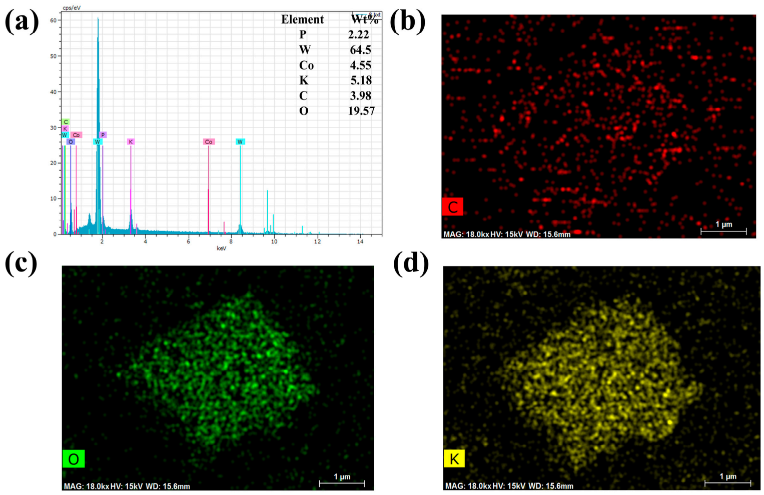

Figure S6. EDS (**a**) and EDX spectra of C (**b**), O (**c**), K (**d**) of {P_2W_{18}}@Co-BTC.

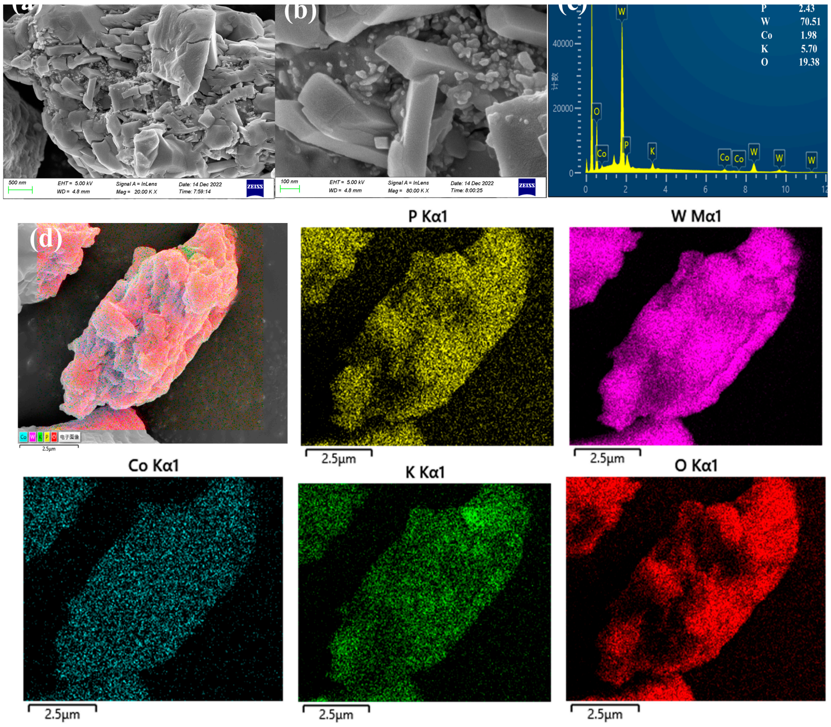

Figure S7. The SEM (**a, b**), EDX (**c**) and mapping (**d**) of $CoK_4[P_2W_{18}O_{62}]$.

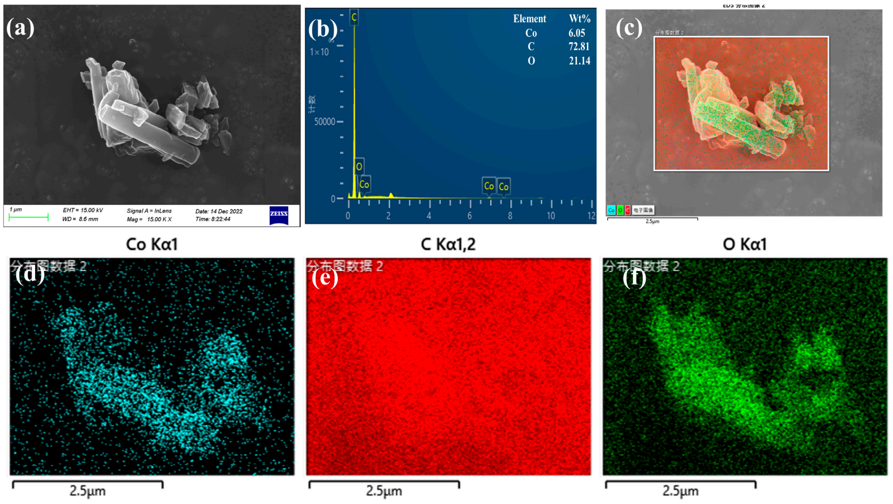

Figure S8. The SEM (**a**), EDS (**b**), EDX (**c**) and mapping (**d**) of Co-BTC. (SEM is directly tested with conductive adhesive as the base, so there are some errors in morphology and content).

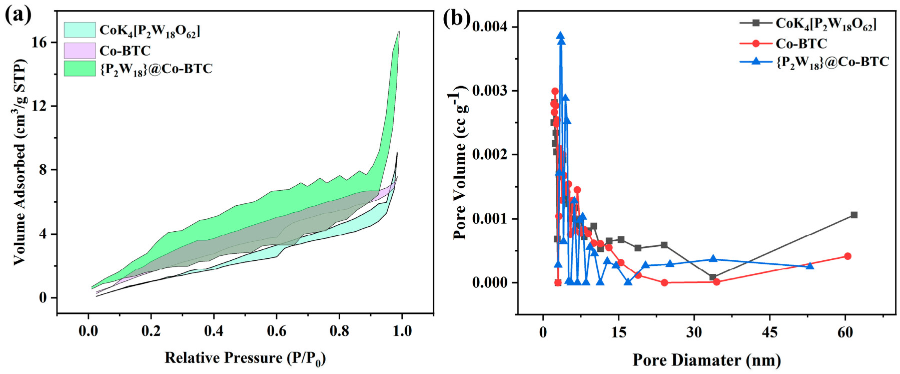

Figure S9. The BET (**a**) and pore size distribution map (**b**) of $\{P_2W_{18}\}$@Co-BTC, $CoK_4[P_2W_{18}O_{62}]$, Co-BTC.

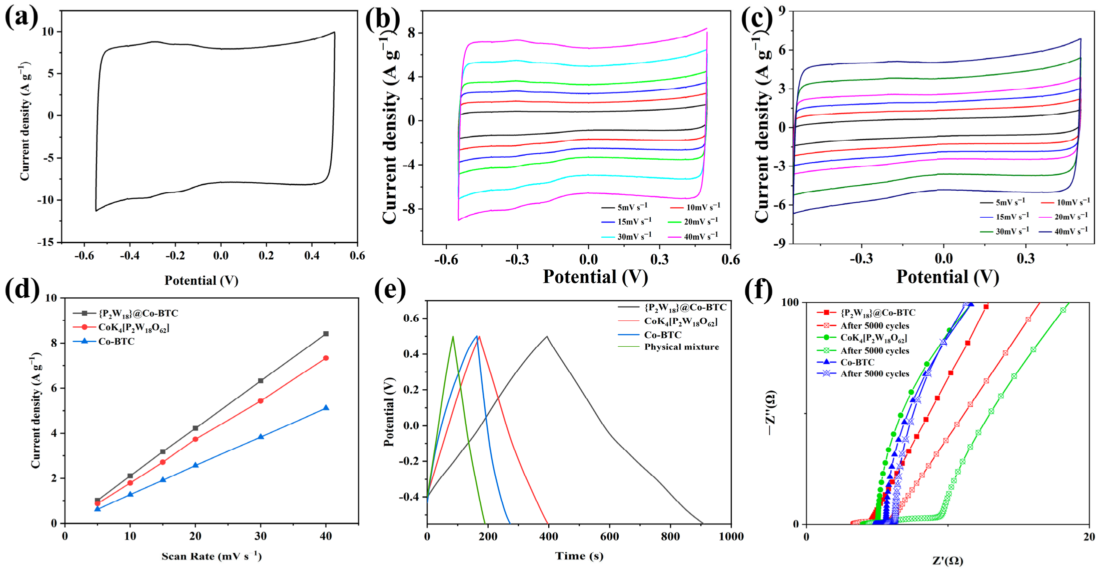

Figure S10. (**a**) The CV of {P_2W_{18}}@Co-BTC in 40 mV s^{-1}; (**b**) CV of $CoK_4[P_2W_{18}O_{62}]$ and (**c**) Co-BTC at three-electrode system; (**d**) The {P_2W_{18}}@Co-BTC, $CoK_4[P_2W_{18}O_{62}]$, Co-BTC plots of the anodic peak currents vs. scan rates; (**e**) Comparison diagram of GCD for {P_2W_{18}}@Co-BTC, $CoK_4[P_2W_{18}O_{62}]$, Co-BTC and physical mixture; (f) The EIS of {P_2W_{18}}@Co-BTC, $CoK_4[P_2W_{18}O_{62}]$, Co-BTC before and after 5000 cycles of the three electrodes system in the high-frequency region.

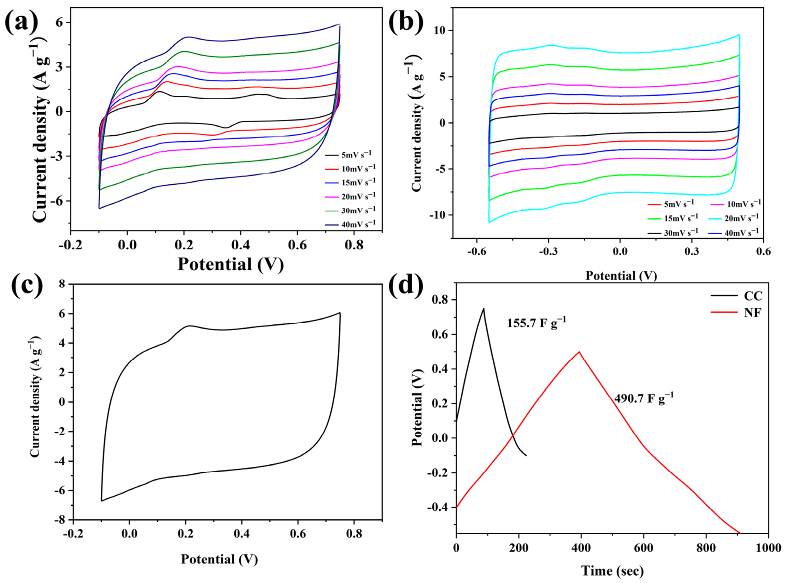

Figure S11. The CV of {P_2W_{18}}@Co-BTC with CC (**a**) and NF (**b**) as collectors; (**c**) The CV of {P_2W_{18}}@Co-BTC in 40 mV s^{-1} with CC as collecter; (**d**) Comparison of GCD under the condition of current density of 1A g^{-1}, CC and NF as collectors.

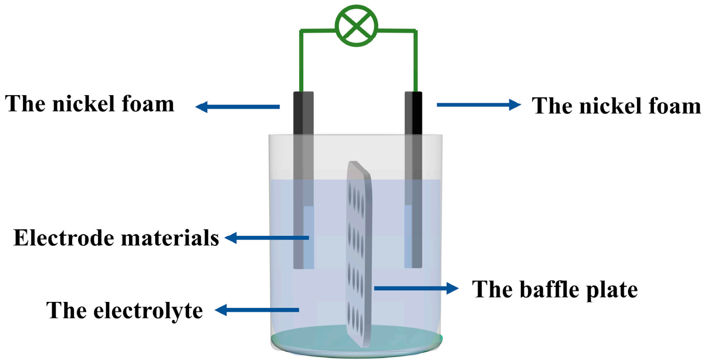

Figure S12. The aqueous symmetric supercapacitor.

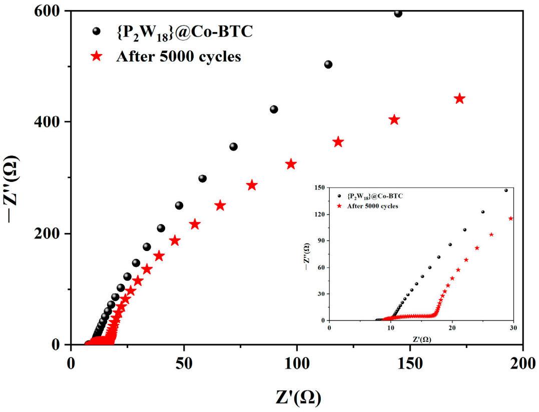

Figure S13. The EIS of {P_2W_{18}}@Co-BTC before and after 5000 cycles of the symmetric double-electrode system (the insert is an enlarged view of the curves in the high-frequency region).

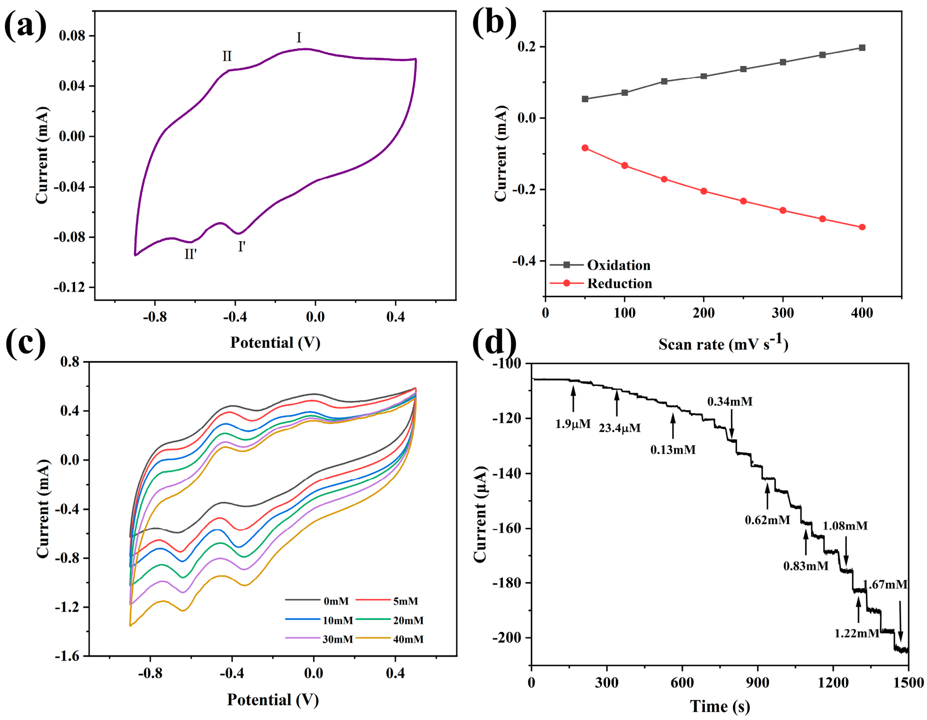

Figure S14. (**a**) The 50 mV s^{-1} CV of {P_2W_{18}}@Co-BTC-GCE ; (**b**) Linear curve of {P_2W_{18}}@Co-BTC-GCE oxidation-reduction peak (II) current as a function of scan rate; (**c**) The CV curves of electrocatalytic reduction of H_2O_2 by {P_2W_{18}}@Co-BTC-GCE; (d) The *i-t* curve of {P_2W_{18}}@Co-BTC-GCE with successive additions of H_2O_2.

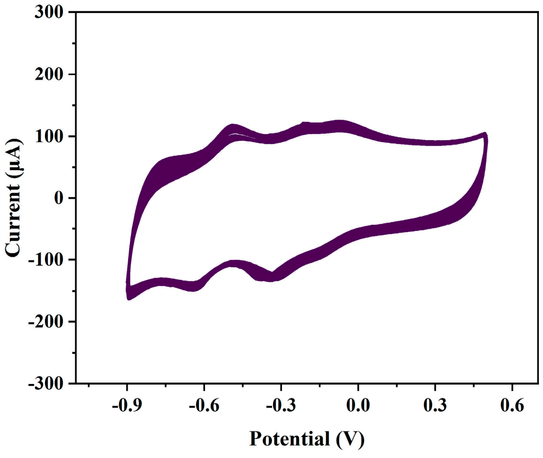

Figure S15. Cyclic stability (1000 cycles) for {P_2W_{18}}@Co-BTC-GCE.

Table S1. {P_2W_{18}} part of the literature summary.

	Compound	Method of synthesis	Performance	Performance values	Ref
1	[{$Cu(btp)_2$}$_3${$As_2Mo_{18}O_{62}$}]	hydrothermal method	photocatalytic and lithium-ion batteries	MB (96%);100 mA·g $^{-1}$ (1050 mAh·g $^{-1}$)	[1]

2	Tris-LDH-P_2W_{18}	"one-pot" anion-exchange method	photocatalytic	MB (83%)	[2]
3	$H_5[Ag_9(trz)_8][P_2W_{18}O_{62}]\cdot 2H_2O$	hydrothermal method	lithium-ion batteries	100 $mA\cdot g^{-1}$ (320 $mAh\cdot g^{-1}$)	[3]
4	$[Cu_8(trz)_6(OH)_4(H_2O)_5][P_2W_{18}O_{62}]\cdot 2H_2O$	hydrothermal method	lithium-ion batteries	100 $mA\cdot g^{-1}$ (420 $mAh\cdot g^{-1}$)	[3]
5	$\{[(Zn(H_2biim)_2)_3(P_2W_{18}O_{62})]_n$	hydrothermal method	enzymeimmobilizing capability for horseradish peroxidase (HRP)	3.11×10^{-3} mmol L^{-1}	[4]
6	ZIF-8/ZnO/$K_6P_2W_{18}O_{62}$	two-step strategy	photocatalytic	MB (96%)	[5]
7	Fe0@PEDO\\P_2W_{18}	solution method	catalytic oxidative desulfurization	35°C/ 60 min (98.4%)	[6]
8	$K_6P_2W_{18}O_{62}$		oxidative decomposition of lignocellulose		[7]
9	P_2W_{18}/Chitosan	one step self-assembly technique	electrocatalysis	stable in pH 1.8 and pH 3.1	[8]
10	$[Cu_6(qx)_6(P_2W_{18}O_{62})(H_2O)_{1.5}]$ • • $4.5H_2O$	hydrothermal method	electrochemistry	reduction of nitrite	[9]
11	$[Ag_6(qx)_6(P_2W_{18}O_{62})]$ • $8H_2O$	hydrothermal method	electrochemistry	reduction of nitrite	[9]
12	HPBs/rGO	the solvent volatilization drive method	electrochemistry	efficiency (8.09%)	[10]
13	$K_6[\alpha\text{-}P_2W_{18}O_{62}]\cdot 14H_2O$		visible light photocatalysis	degradation of MB	[11]
14	P_2W_{18} • NiO@TiO_2	calcination method	dye-sensitized solar cells	τe valve 8.92ms	[12]
15	P_2W_{18} • CuO@TiO_2	calcination method	dye-sensitized solar cells	τe valve 5.53ms	[12]
16	$[Cu(phen)_2][Cu(phen)(H_2O)_3][Cu(phen)_2(H_2O)][P_2W_{18}O_{62}]\cdot 8H_2O$	hydrothermal method	photocatalytic	styrene conversion (78.4%)	[13]
17	$[Cu(2,2'\text{-}bpy)(H_2O)_2]_2[Cu(2,2'\text{-}bpy)_2][P_2W_{18}O_{62}]\cdot 3.5H_2O$	hydrothermal method	photocatalytic	styrene conversion (54.0%)	[13]
18	$[Ag(2,2'\text{-}bpy)_2]_5[HP_2W_{18}O_{62}]\cdot H_2O$	hydrothermal method	photocatalytic	styrene conversion (23.8%)	[13]
19	$[Ag(2,2'\text{-}bpy)(H_2O)]_2[Ag(2,2'\text{-}bpy)]_2[Ag_2(2,2'\text{-}bpy)_3][P_2W_{18}O_{62}]$	hydrothermal method	photocatalytic	styrene conversion (21.3%)	[13]
20	$[Ag(2,2'\text{-}bpy)(H_2O)]_3[Ag_2(2,2'\text{-}bpy)_3][Ag(2,2'\text{-}bpy)][P_2W_{18}O_{62}]\cdot H_2O$	hydrothermal method	photocatalytic	styrene conversion (24.4%)	[13]

21	$[H_6P_2W_{18}O_{62}](phen)_{5.5}$	hydrothermal method	photocatalytic	styrene conversion (12.0%)	[13]
22	$[H_6P_2W_{18}O_{62}](phen)_3{\cdot}43H_2O$	hydrothermal method	photocatalytic	styrene conversion (13.1%)	[13]
23	$[P_2W_{18}/CS\text{-}CNTs]_n$	layer-bylayer (LbL) assembly method	Electrochromic	optical contrast (20.3%)	[14]
24	$(C_4H_{10}N)_6\ (P_2Mo_{18}O_{62}) \bullet 4H_2O$	Backflow and hydrothermal method	glucose biofuel cell anodes and glucose biosensors	0.2 V (4.7 mA cm^{-2})	[15]
25	$(C_6H_8NO)_4\ [H_2P_2W_{18}O_{62}] \bullet 6H_2O$	Backflow and hydrothermal method	glucose biofuel cell anodes and glucose biosensors	0.2 V (0.34 mA cm^{-2})	[15]
26	$(P_2W_{18}\text{-}SnO_2\text{-}AuNPs)_3$	layer-by-layer self-assembly strategy	electrochemical detection of myricetin	recovery range（97.65%~103.07%）	[16]
27	AC/P_2Mo_{18}		supercapacitor	308 F g^{-1} (2 A g^{-1})	[17]
28	$(H_2bpe)(Hbpe)_2\{[Cu(pzta)(H_2O)][P_2W_{18}O_{62}]\}{\cdot}5H_2O$	hydrothermal method	supercapacitor and reduction of H_2O_2 and KIO_3	168 F g^{-1} (5 A g^{-1})	[18]
29	$(Cu(biz)_2)_{12}[Cu^I(H_2O)_2\{As_{0.5}{}^{III}\ As_2Mo^V{}_2Mo^{VI}{}_{16}O_{62}\}_2]{\cdot}2H_2O$	hydrothermal method	electrocatalyst and photocatalyst	MB (97.29%)	[19]
30	$(biz)_6(ptz)\ \{As^{III}{}_2As_2Mo_{18}O_{62}\}{\cdot}H_2O$	hydrothermal method	electrocatalyst and photocatalyst	MB (95.52%)	[19]
31	$(H_2bimb)_2[\{Ag_{1.5}(bimb)_{1.5}\}(P_2W_{18}O_{62})]$	hydrothermal method	electrocatalyst and photocatalyst	MB (93.5%)	[20]
32	$(H_2bimb)_2[Ag(bimb)]_{0.5}[Ag(eim)_2]_{0.5}[P_2W_{18}O_{62}]{\cdot}H_3O$	hydrothermal method	electrocatalyst and photocatalyst	MB (94.2%)	[20]
33	$(H_2bip)_2\{H_2P_2W_{18}O_{62}\}{\cdot}2H_2O$	hydrothermal method	supercapacitor and electrochemical sensor	802.4 F g^{-1} (3 A g^{-1})	[21]
34	$(bipy)_{0.5}\{Ag(bipy)_2\}\{Ag_4(bipy)_7\}\{NaP_2W_{18}O_{62}\}{\cdot}H_2O$	hydrothermal method	supercapacitor and electrochemical sensor	752.1 F g^{-1} (3 A g^{-1})	[21]
35	$(H_2bib)_{2.5}\{HP_2Mo_{18}O_{62}\}{\cdot}2H_2O$	hydrothermal method	supercapacitor and electrochemical sensor	573.7 F g^{-1} (3 A g^{-1})	[22]
36	$(Hbipy)_2[Mn(bipy)_3]_2[As_2W_{18}O_{62}]$	hydrothermal method	supercapacitor and hydrogen evolution reaction	789.5 F g^{-1} (3 A g^{-1})	[23]

37	$[\{Cu^{II}(bim)_2\}_3(As_2W_{18}O_{62})]$	hydrothermal method	supercapacitor and electrochemical sensor	834.8 (2.4 A g^{-1})	[24]
38	$[(Cu^{I}_{10}pz_{10}Cl_4)(As_2W_{18}O_{62})]$	hydrothermal method	supercapacitor and electrochemical sensor	960.1 F g^{-1} (2.4 A g^{-1})	[24]
39	$\{Ag_4K_2P_2W_{18}O_{62}\}$@$[Ag_3(\mu$-Hbtc)($\mu$-$H_2$btc)$]_n$	liquid-assisted grinding	supercapacitor and H_2O_2 sensor	378.9 F g^{-1} (1 A g^{-1})	[25]
40	$[\{K(H_2O)\}_2\{Cu_2(bim)_2\}_2(P_2W_{18}O_{62})]$	hydrothermal method	supercapacitor and H_2O_2 sensor	1070 F g^{-1} (2 A g^{-1})	[26]
41	$[Ru(bpy)_3]_{3.33}P_2Mo_{18}O_{62}\cdot mH_2O$	solution method	supercapacitor	125 F g^{-1} (0.2 A g^{-1})	[27]
42	$[Ru(bpy)_3]_3P_2Mo_{18}O_{62}\cdot nH_2O$	solution method	supercapacitor	68 F g^{-1} (0.2 A g^{-1})	[27]

Table S2. Atomic percent (%) of corresponding samples by ICP-MS analysis.

	**P**	**K**	**Co**	**W**
$\{P_2W_{18}\}$@Co-BTC	0.88	4.95	3.01	8.89

Table S3. Performance comparison of Dawson -based materials with published polyoxometalate supercapacitors.

	**materials**	**specific capacitance**	**cycling stability**	**current collector**	**Ref.**
1	AC/P_2Mo_{18}	308 F g^{-1} (2 A g^{-1})	70% (2000 cycles)		[17]
2	$(H_2bpe)(Hbpe)_2\{[Cu(pzta)(H_2O)][P_2W_{18}O_{62}]\}\cdot 5H_2O$	168 F g^{-1} (5 A g^{-1})	90.7% (1000 cycles)	glassy carbon	[18]
3	$(H_2bip)_2\{H_2P_2W_{18}O_{62}\}\cdot 2H_2O$	802.4 F g^{-1} (3 A g^{-1})	93.6% (5000 cycles)	Carbon Paper	[21]
4	$(bipy)_{0.5}\{Ag(bipy)_2\}\{Ag_4(bipy)_7\}\{NaP_2W_{18}O_{62}\}\cdot H_2O$	752.1 F g^{-1} (3 A g^{-1})	94.8% (5000 cycles)	Carbon Paper	[21]
5	$(H_2bib)_{2.5}\{HP_2Mo_{18}O_{62}\}\cdot 2H_2O$	573.7 F g^{-1} (3 A g^{-1})	85.7% (5000 cycles)	glassy carbon	[22]
6	$(Hbipy)_2[Mn(bipy)_3]_2[As_2W_{18}O_{62}]$	789.5 F g^{-1} (3 A g^{-1})	90.6% (5000 cycles)	Carbon Paper	[23]
7	$[\{Cu^{II}(bim)_2\}_3(As_2W_{18}O_{62})]$	834.8 (2.4 A g^{-1})	89.3% (5000 cycles)	glassy carbon	[24]
8	$[(Cu^{I}_{10}pz_{10}Cl_4)(As_2W_{18}O_{62})]$	960.1 F g^{-1} (2.4 A g^{-1})	91.9% (5000 cycles)	glassy carbon	[24]
9	$\{Ag_4K_2P_2W_{18}O_{62}\}$@$[Ag_3(\mu$-Hbtc)($\mu$-$H_2$btc)$]_n$	378.9 F g^{-1} (1 A g^{-1})	93.5 % (5000 cycles)	nickel foam	[25]
10	$[\{K(H_2O)\}_2\{Cu_2(bim)_2\}_2(P_2W_{18}O_{62})]$	1070 F g^{-1} (2 A g^{-1})	89.8% (5000 cycles)	glassy carbon	[26]
11	$[Ru(bpy)_3]_{3.33}P_2Mo_{18}O_{62}\cdot mH_2O$	125 F g^{-1} (0.2 A g^{-1})	87% (500 cycles)	glassy carbon	[27]
12	$[Ru(bpy)_3]_3P_2Mo_{18}O_{62}\cdot nH_2O$	68 F g^{-1}	57%	glassy carbon	[27]

		(0.2 A g^{-1})	(500 cycles)		
13	$[Ag_{10}(trz)_6][SiW_{12}O_{40}]$	47.8 F g^{-1} (1.5 A g^{-1})	90.9% (1000 cycles)	glassy carbon	[28]
14	$[Ag_{10}(trz)_8][HVW_{12}O_{40}]$	93.5 F g^{-1} (1.5 A g^{-1})	59.2% (750 cycles)	glassy carbon	[28]
15	$[Cu^I(btx)]_4[SiW_{12}O_{40}]$	110.3 F g^{-1} (3 A g^{-1})	87% (1000 cycles)	glassy carbon	[29]
16	$PAni/H_3PMo_{12}O_{40}$	120 F g^{-1} (0.4 A g^{-1})	70% (1000 cycles)	Rigid graphite plate	[30]
17	$rGO/PMo_{12}O_{40}$	276 F g^{-1} (10 mV s^{-1})	96% (10000 cycles)	Graphite roods	[31]
18	$AC/PMo_{12}O_{40}$	223 F g^{-1} (1 mV s^{-1})	100% (10000 cycles)	Ti foils	[32]
19	Pinecone $AC/PMo_{12}O_{40}$	361 F g^{-1} (10 mV s^{-1})		titanium foil	[33]
20	$rGO\text{-}PMo_{12}$	269 mF cm^{-2} (5 mA cm^{-2})	89% (5000 cycles)	carbon cloth	[34]
21	$PMo_{10}V_2$@ZIF-67	475 F g^{-1} (2 A g^{-1})	106.41% (5000 cycles)	Ni foam	[35]
22	$AC/TEAPW_{12}$	82 F g^{-1} (0.5 A g^{-1})	93% (10000 cycles)	aluminum foil	[36]
23	PW_{12}@MIL-101	158 mF·cm^{-2} (0.5 mA·cm^{-2})		nickel foam	[37]
24	$P_{03}S_{02}\text{-}PMo_{12}$	205 F g^{-1} (1 mV s^{-1})			[38]
25	$P_{04}S_{30}\text{-}PMo_{12}$	282 F g^{-1} (1 mV s^{-1})			[38]
26	$P_{04}S_{60}\text{-}PMo_{12}$	326 F g^{-1} (1 mV s^{-1})			[38]
27	$[Co^{II}(pzta)_2(H_2O)]_2(H_4GeMo_{12}O_{40})\cdot 4H_2O$	182 F g^{-1} (1 A g^{-1})	86% (1000 cycles)		[39]
28	$[Co^{II}(pzta)_2(H_2O)]_2(H_4SiMo_{12}O_{40})\cdot 4H_2O$	121.5 F g^{-1} (1 A g^{-1})	91% (1000 cycles)		[39]
29	$[Cu_3(bty)_3][BW_{12}O_{40}]\cdot 4H_2O$	214.59 F g^{-1} (0.48 A g^{-1})	91.55% (1000 cycles)	carbon cloth	[40]
30	$[Cu_{2.5}(bty)_5][BW_{12}O_{40}]\cdot 7H_2O$	189.17 F g^{-1} (0.48 A g^{-1})	90.2% (1000 cycles)	carbon cloth	[40]
31	$[Cu_2(H_2O)4H_2(imbta)_4](PMo_{12}O_{40})_2\cdot 6H_2O$	710 F g^{-1} (1 A g^{-1})	91.2% (1000 cycles)	glassy carbon	[41]
32	$[Cu(H_2O)_2H_4(pybta)_4](PMo_{12}O_{40})_2\cdot 2H_2O$	556F g^{-1} (1 A g^{-1})	92.2% (1000 cycles)	glassy carbon	[41]
33	$[Ag_2H_7(pybta)_6(PMo_{12}O_{40})_3]\cdot 12H_2O$	408 F g^{-1} (1 A g^{-1})	86.2% (1000 cycles)	glassy carbon	[41]
34	$[AgH_2(imbta)_2](PMo_{12}O_{40})$	320 F g^{-1} (1 A g^{-1})	70.0% (1000 cycles)	glassy carbon	[41]
35	$\{P_2W_{18}\}$@Co-BTC	490.7 F g^{-1} (1 A g^{-1})	90.6% (5000 cycles)	nickel foam (three electrodes)	This work

References

1. Cong, B.W.; Su, Z.H.; Zhao, Z.F.; Wang, B. A novel 3D POMOFs based on Wells-Dawson arsenomolybdates with excellent photocatalytic and lithium-ion batteries performance. CrystEngComm **2017**, 19, 7154-7161.
2. Chen, Y.; Yao, Z.X.; Miras, H.N.; Song Y.F. Modular Polyoxometalate-Layered Double Hydroxide Composites as Efficient Oxidative Catalysts. Chem. Eur. J. **2015**, 21, 10812 – 10820.
3. Yang, X.Y.; Li, M.T.; Sheng, N.; Li, J.S.; Liu, G.D.; Sha, J.Q.; Jiang, J.Z. Structure and LIBs Anode Material Application of Novel Wells-Dawson Polyoxometalate-based Metal Organic Frameworks with Different Helical Channels. Cryst. Growth Des. **2018**, 18, 5564–5572.
4. Zhu, H.T.; Du, J.; Lu, Y.; Su, F.; Li, Y.G. Immobilization of enzymes on an organic–inorganic hybrid network consisting of Dawson-type polyoxotungstate and a zinc(II)-biimidazole complex moiety. New J. Chem. **2019**, 43, 146.
5. Hong, B.; Liu, L.; Wang, S.M.; Han, Z.B. Facile Synthesis of ZIF-8/ZnO/Polyoxometalate Ternary Composite Materials for Efficient and Rapid Removal of Cationic Organic Dye. J. Clust. Sci. **2016**, 27, 563–571.
6. Ammar, S.H.; Salman, M.D.; Shafi, R.F. Keggin- and Dawson-type polyoxotungstates immobilized on poly(3, 4-ethylenedioxythiophene)-coated zerovalent iron nanoparticles: synthesis, characterization and their catalytic oxidative desulfurization activity. J. Environ. Chem. Eng. **2021**, 9, 104904.
7. Shi, N.; Liu, D.; Dang, F.L.; Chen, Q.T.; Li, M.; Wen, F.S. Bifunctional and recyclable Dawson-type polyoxometalates catalyze oxidative degradation of lignocellulose to selectively produce phthalates. Bioresource Technol. **2019**, 273, 677-681.
8. Shan, Y.P.; Yang, G.C.; Jia, Y.T.; Gong, J.; Su, Z.M.; Qu, L.Y. ITO electrode modified with chitosan nanofibers loading polyoxometalate by one step self-assembly method and its electrocatalysis. Electrochem. Commun. **2007**, 9, 2224–2228.
9. Chi, Y.N.; Cui, F.Y.; Lin, Z.G.; Xu, Y.; Ma, X.Y.; Shen, P.P.; Huang, K.L.; Hu, C.W. Assembly of Cu/Ag-quinoxaline-polyoxotungstate hybrids: Influence of Keggin and Wells–Dawson polyanions on the structure. J. Solid State Chem. **2013**, 199, 230-239.
10. Li, J.P.; Wu, D.; Wang, C.L.; Liu, D.; Chen, W.L.; Wang, X.L.; Wang, E.B. Interfacial self-assembly engineering for constructing a 2D flexible superlattice polyoxometalate/rGO heterojunction for high-performance photovoltaic devices. Dalton Trans. **2020**, 49, 3766-3774.
11. Wang, R.X.; Liu, Y.F.; Zuo, P.; Zhang, Z.D.; Lei, N.N.; Liu, Y.Q. Phthalocyanine-sensitized evolution of hydrogen and degradation of organic pollutants using polyoxometalate photocatalysts. Environ. Sci. Pollut. Res. **2020**, 27, 18831–18842.
12. Zheng, X.T.; Chen, W.L.; Chen, L.; Wang, Y.J.; Guo, X.W.; Wang, J.B.; Wang, E.B. A strategy for breaking polyoxometalate-based MOFs to obtain high loading amount of nanosized polyoxometalate clusters to improve the performance of dye-sensitized solar cells. Chem. Eur. J. **2017**, 23, 8871-8878.
13. Lü, Y.; Xiao, L.N.; Hao, X.R.; Cui, X.B.; Xu, J.Q. A series of organic–inorganic hybrid compounds formed by $[P_2W_{18}O_{62}]^{6-}$ and several types of transition metal complexes. Dalton Trans. **2017**,46, 14393-14405.
14. Liu, S.P.; Xu, L.; Li, F.Y.; Guo, W.H.; Xing, Y.; Sun, Z.X. Carbon nanotubes-assisted polyoxometalate nanocomposite film with enhanced electrochromic performance. Electrochim. Acta **2011**, 56, 8156-8162.
15. Boussema, F.; Grossa, A.J.; Hmida, F.; Ayed, B.; Majdou, H.; Cosniera, S.; Maaref, A.; Holzingera, M. Dawson-type polyoxometalate nanoclusters confined in a carbon nanotube matrix as efficient redox mediators for enzymatic glucose biofuel cell anodes and glucose biosensors. Biosens. Bioelectron. **2018**, 109, 20-26.
16. Xing, R.M.; Tong, L.Y.; Zhao, X.Y.; Liu, H.L.; Ma, P.T.; Zhao, J.W.; Liu, X.Q.; Liu, S.H. Rapid and sensitive electrochemical detection of myricetin based on polyoxometalates/SnO_2/gold nanoparticles ternary nanocomposite film electrode. Sens. Actuators B Chem. **2019**, 283, 35-41.
17. Mu, A.Q.; Li, J.S.; Chen, W.L.; Sang, X.J.; Su, Z.M.; Wang, E.B. The composite material based on Dawson-type polyoxometalate and activated carbon as the supercapacitor electrode. Inorg. Chem. Commun. **2015**, 55, 149-152.
18. Wang, G.N.; Chen, T.T.; Li, S.B.; Pang, H.J.; Ma, H.Y. A coordination polymer based on dinuclear (pyrazinyl tetrazolate) copper(ii) cations and Wells–Dawson anions for high-performance supercapacitor electrodes. Dalton Trans. **2017**, 46, 13897-13902.
19. Guan, Y.; Cui, L.P.; Yu, K.; Lv, J.H.; Deng, Y.F.; Wang, C.M.; Zhou, B.B. Two arsenic capped Dawson-type supramolecular hybrid assemblies induced by benzimidazole for photo-/electro-catalytic performance. J. Solid State Chem. **2020**, 292, 121707.
20. Fan, X.Y.; Guo, H.; Lv, J.H.; Yu, K.; Su, Z.H.; Wang, L.; Wang, C.M.; Zhou, B.B. Efficient and robust photocatalysts based on $\{P_2W_{18}\}$ modified by Ag complex. Dalton Trans. **2018**, 47, 4273-4281.
21. Yang, A.S.; Cui, L.P.; Yu, K.; Lv, J.H.; Ma, Y.J.; Zhao, T.T.; Zhou, B.B. Supramolecular Host−Guest Assembly Based on Phosphotungstate Nanostructures for Pseudocapacitive and Electrochemical Sensing Applications. ACS Appl. Nano Mater. **2022**, 5, 10452−10461.
22. He, L.L.; Cui, L.P.; Yu, K.; Lv, J.H.; Ma, Y.J.; Tian, R.; Zhou, B.B. The pseudocapacitance and sensing materials constructed by Dawson/basket-like phosphomolybdate. J. Solid State Chem. **2022**, 316, 123578.
23. Tian, R.; Cui, L.P.; Yu, K.; Lv, J.H.; Ma, Y.J.; He, L.L.; Zhou, B.B. Arsenotungstate-Nanostructure-Based Derivatives with One-Dimensional Tunnels for Electrochemical Capacitors and Electrocatalytic Hydrogen Evolution. ACS Appl. Nano Mater. **2022**, 5, 14882−14892.
24. Zhong, R.; Cui, L.P.; Yu, K.; Lv, J.H.; Guo, Y.H.; Zhang, E.M.; Zhou, B.B. Wells−Dawson Arsenotungstate Porous Derivatives for Electrochemical Supercapacitor Electrodes and Electrocatalytically Active Materials. Inorg. Chem. **2021**, 60, 9869−9879.
25. Liang, Y.; Di, S.; Wang, C.M.; Yu, K.; Wang, C.X.; Lv, J.H.; Zhou, B.B. Synthesis of $\{P_2W_{18}\}$-based coated structured nano materials with supercapacitors and H_2O_2 sensing. J. Energy Storage **2022**, 56, 105991.

26. Gao, J.Q.; Gong, L.G.; Fan, X.Y.; Yu, K.; Zheng, Z.W.; Zhou, B.B. {$P_2W_{18}O_{62}$}-Encapsulated Potassium-Ion Nanotubes Intercalated in Copper Biimidazole Frameworks for Supercapacitors and Hydrogen Peroxide Sensing. ACS Appl. Nano Mater. 2020, 3, 1497-1503.
27. Chinnathambi, S.; Ammam, M. A molecular hybrid polyoxometalate-organometallic moieties and its relevance to supercapacitors in physiological electrolytes Selvaraj Chinnathambi, J. Power Source **2015**, 284, 524-535.
28. Hou, Y.; Pang, H.J.; Gómez-García, C.J.; Ma, H.Y.; Wang, X.M.; Tan, L.C. Polyoxometalate Metal−Organic Frameworks: Keggin Clusters Encapsulated into Silver-Triazole Nanocages and Open Frameworks with Supercapacitor Performance. Inorg. Chem. **2019**, 58, 16028-16039.
29. Chai, D.F.; Hou, Y.; O'Halloran, K.P.; Pang, H.J.; Ma, H.Y.; Wang, G.N.; Wang, X.M. Enhancing Energy Storage via TEA-Dependent Controlled Syntheses: Two Series of Polyoxometalate-Based Inorganic-Organic Hybrids and their Supercapacitor Properties. Chem. Electro. Chem. **2018**, 5, 3443-3450.
30. Gallegos, A.K.C.; Cantú, M.L.; Pastor, N.C.; Romero, P.G. Nanocomposite Hybrid Molecular Materials for Application in Solid-State Electrochemical Supercapacitors. Adv. Funct. Mater. **2015**, 15, 1125-1133.
31. Guevara, J.S.; Ruiza, V.; Romero, P.G. Stable graphene–polyoxometalate nanomaterials for application in hybrid supercapacitors. Phys. Chem. Chem. Phys. **2014**, 16, 20411-20414.
32. Hu, C.C.; Zhao, E.B.; Nitta, N.; Magasinski, A.; Berdichevsky, G.; Yushin, G. Aqueous solutions of acidic ionic liquids for enhanced stability of polyoxometalate-carbon supercapacitor electrodes. J. Power Sources **2016**, 326, 569-574._
33. Genovese, M.; Lian, K. Polyoxometalate modified pine cone biochar carbon for supercapacitor electrodes. J. Mater. Chem. A **2017**, 5, 3939-3947.
34. Dubal, D.P.; Chodankar, N.R.; Vinu, A.; Kim, D.H.; Gomez-Romero, P. Asymmetric Supercapacitors Based on Reduced Graphene Oxide with Different Polyoxometalates as Positive and Negative Electrodes. ChemSusChem **2017**, 10, 2742-2750.
35. Mohamed, A.M.; Ramadan, M.; Ahmed, N.; ElNaga, A.O.A.; Alalawy, H.H.; Zaki, T.; Shaban, S.A.; Hassan, H.B.; Allam, N.K. Metal–Organic frameworks encapsulated with vanadium-substituted heteropoly acid for highly stable asymmetric supercapacitors. J. Energy Storage **2020**, 28, 101292.
36. Zhu, J.J.; Vilaua, R.B.; Romero, P.G. Can polyoxometalates enhance the capacitance and energy density of activated carbon in organic electrolyte supercapacitors? Electrochim. Acta **2020**, 362, 137007.
37. Li, T.Y.; He, P.; Dong, Y.N.; Chen, W.C.; Wang, T.; Gong, J.; Chen, W.L. Polyoxometalate-Based Metal-Organic Framework/Polypyrrole Composites toward Enhanced Supercapacitor Performance. Eur. J. Inorg. Chem. **2021**, 21, 2063-2069.
38. Quezada, E.F.; Casillas, D.C.M.; Gallegos, A.K.C.; Llave, E.d.l. Effect of Hierarchical Porosity on PMo_{12} Adsorption and Capacitance in Hybrid Carbon–PMo_{12} Electrodes for Supercapacitors. Energy Fuels **2022**, 36, 3987–3996.
39. Hu, S.M.; Li, K.Q.; Yu, X.J.; Jin, Z.X.; Xiao, B.X.; Yang, R.R.; Pang, H.J.; Ma, H.Y.; Wang, X.M.; Tan, L.C.; Yang G.X. Enhancing the electrochemical capacitor performance of Keggin polyoxometalates by anchoring cobalt-triazole complexes. J. Mol. Struct. **2022**, 1250, 131753.
40. Hou, Y.J.; Han, P.L.; Zhang, L.K.; Li H.; Xu Z.H. pH-controlled assembling of POM-based metal–organic frameworks for use as supercapacitors andefficient oxidation catalysts for various sulfides. Inorg. Chem. Front. **2023**, 10, 148-157.
41. Yu, X.J.; Khan, S.U.; Jin, Z.X.; Wu, Q.; Pang, H.J.; Ma, H.Y.; Wang, X.M.; Tan, L.C.; Yang, G.X. Water cluster encapsulated polyoxometalate-based hydrogen-bonded supramolecular frameworks (PHSFs) as a new family of high-capacity electrode materials. J. Energy Storage **2022**, 53, 105192.

Disclaimer/Publisher's Note: The statements, opinions and data contained in all publications are solely those of the individual author(s) and contributor(s) and not of MDPI and/or the editor(s). MDPI and/or the editor(s) disclaim responsibility for any injury to people or property resulting from any ideas, methods, instructions or products referred to in the content.
